# A non-conducting role of the Ca_v_1.4 Ca^2+^ channel drives homeostatic plasticity at the cone photoreceptor synapse

**DOI:** 10.1101/2023.12.05.570129

**Published:** 2023-12-06

**Authors:** J. Wesley Maddox, Gregory J. Ordemann, Juan de la Rosa Vázquez, Angie Huang, Christof Gault, Serena R. Wisner, Kate Randall, Daiki Futagi, Steven H. DeVries, Mrinalini Hoon, Amy Lee

**Affiliations:** 1 Dept of Neuroscience, University of Texas-Austin, Austin, TX 78712, USA; 2 Dept. of Ophthalmology and Visual Sciences, University of Wisconsin- Madison, Madison, WI, 53706, USA; 3 Neuroscience Training Program, University of Wisconsin-Madison, Madison WI 53706 USA; 4 Dept. of Ophthalmology, Northwestern University Feinberg School of Medicine, Chicago, IL, 60611, USA; 5 McPherson Eye Research Institute, Madison WI 53706 USA; 6 These authors contributed equally

**Keywords:** photoreceptor, Ca^2+^ channel, ribbon synapse, retina, synaptogenesis

## Abstract

In congenital stationary night blindness type 2 (CSNB2)—a disorder involving dysfunction of the Ca_v_1.4 Ca^2+^ channel—visual impairment is relatively mild considering that Ca_v_1.4 mediates synaptic transmission by rod and cone photoreceptors. Here, we addressed this conundrum using a Ca_v_1.4 knockout (KO) mouse and a knock-in (KI) mouse expressing a non-conducting Ca_v_1.4 mutant. Surprisingly, aberrant Ca_v_3 currents were detected in cones of the KI and KO but not wild-type mice. Cone synapses, which fail to develop in KO mice, are present but enlarged in KI mice. Moreover, light responses in cone pathways and photopic visual behavior are preserved in KI but not in KO mice. In CSNB2, we propose that Ca_v_3 channels maintain cone synaptic output provided that the Ca^2+^-independent role of Ca_v_1.4 in cone synaptogenesis remains intact. Our findings reveal an unexpected form of homeostatic plasticity that relies on a non-canonical role of an ion channel.

## INTRODUCTION

At the first synapse in the visual pathway, the light-dependent graded electrical signals produced in rod and cone photoreceptors gates the release of glutamate onto postsynaptic neurons. To accomplish this task, photoreceptor synapses are specialized with a ribbon organelle, which helps prime synaptic vesicles^1, 2^ and postsynaptic dendrites from horizontal and bipolar cells that invaginate deep within the terminal^3^. A variety of proteins interact with the ribbon and synaptic vesicles near release sites (i.e., active zones)^4^. The importance of these proteins for vision is illustrated by the numerous inherited retinal diseases linked to mutations in their encoding genes^5^.

One such gene is *CACNA1F*, which encodes the voltage-gated Ca^2+^ (Ca_v_) channel expressed in retinal photoreceptors, Ca_v_1.4^6–8^. Among the sub-family of Ca_v_1.x L-type channels, Ca_v_1.4 exhibits unusually slow inactivation that is well-matched for supporting the tonic, Ca^2+^-dependent release of glutamate from photoreceptor synaptic terminals in darkness^9, 10^. More than 200 mutations in *CACNA1F* cause vision disorders including congenital stationary night blindness type 2 (CSNB2)^11, 12^. These mutations are broadly categorized as producing a gain of function or loss of function in Ca_v_1.4^13^. How these mutations in *CACNA1F* lead to the variable clinical phenotypes of CSNB2 is largely unknown. Symptoms may include strabismus, low visual acuity, and in many cases, night blindness^14, 15^. The latter suggests a primary defect in rod pathways, which is surprising given that knockout (KO) mice are completely blind and lack any evidence of either rod or cone synaptic responses^6, 7, 16^. A major caveat is that rod and cone synapses fail to form in Ca_v_1.4 KO mice^7, 16–18^. Thus, Ca_v_1.4 KO mice are not suitable for studies of how *CACNA1F* mutations differentially affect rod and cone pathways or for efforts to uncover how the biophysical properties of Ca_v_1.4 shape photoreceptor synaptic release properties.

Here, we overcome this hurdle with a knock-in mouse strain (G369i KI) expressing a non-conducting mutant form of Ca_v_1.4^19^. We show that cone ribbon synapses in G369i KI mice are largely preserved and that downstream signaling through cone pathways, although greatly impaired, can support visual function. This novel mechanism requires the ability of the Ca_v_1.4 protein, independent of its Ca^2+^ conductance, to nucleate the assembly of cone ribbon synapses and involves an aberrant Ca_v_3 (T-type) conductance that appears when Ca_v_1.4 Ca^2+^ signals are compromised.

## RESULTS

### Ca^2+^ currents in cones are mediated by Ca_v_3 channels upon Ca_v_1.4 loss-of-function

**Figure 1.**
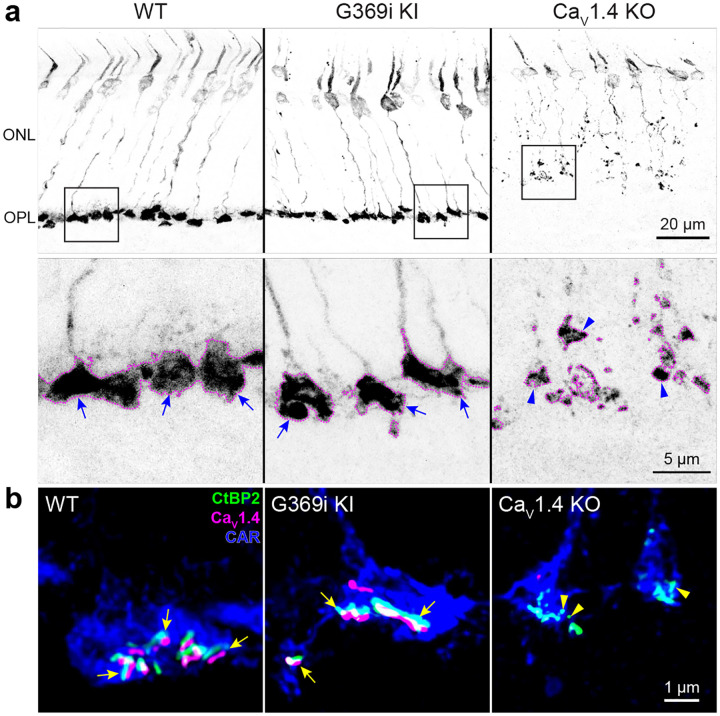
Cone pedicles and the mutant Ca_v_1.4 channel are normally localized in the OPL of G369i KI mice but not Ca_v_1.4 KO mice. Confocal images of the ONL and OPL of WT, G369i KI, and Ca_v_1.4 KO mice labeled with antibodies against cone arrestin (CAR), CtBP2, and Ca_v_1.4. ***a***, Inverted images of CAR labeling. Lower panels correspond to boxed region of the upper panels and depict pedicles labeled by CAR antibodies (dotted outlines). Cone pedicles remain within the OPL of WT and G369i KI retina (arrows) but are misshapen and retracted in the ONL of the Ca_V_1.4 KO retina (arrowheads). ***b***, High magnification, deconvolved images show Ca_V_1.4 labeling near cone ribbons in WT and G369i KI pedicles (arrows) and ribbon spheres without Ca_V_1.4 labeling in the Ca_V_1.4 KO pedicle (arrowheads).

A prevailing yet unsupported hypothesis regarding the relatively mild visual phenotypes in CSNB2 is that additional Ca_v_ subtypes may compensate for Ca_v_1.4 loss of function in cones. If so, then Ca^2+^ currents (*I*_*Ca*_) mediated by these subtypes should be evident in cones of Ca_v_1.4 KO and G369i KI mice. The G369i mutation is an insertion of a glycine residue in a transmembrane domain, which prevents Ca^2+^ permeation through the channel^19^. Rods of G369i KI mice lack any evidence of *I*_*Ca*_, despite the normal presynaptic clustering of the mutant channel^19^. To test if this might differ in G369i KI cones, we first analyzed the localization of the mutant G369i Ca_v_1.4 channels in cones by immunofluorescence with antibodies against Ca_v_1.4, as well as cone arrestin (CAR) and CtBP2 to label cone terminals (i.e., pedicles) and ribbons, respectively (Fig. 1). In Ca_v_1.4 KO mouse retinas, cone pedicles were shrunken and retracted into the outer nuclear layer (ONL, Fig. 1a) and had malformed ribbons (Fig. 1b). In contrast, cone pedicles in G369i KI mice were normally localized in the outer plexiform layer (OPL, Fig. 1a) and were populated by multiple ribbons (Fig. 1b). As in WT mice, labeling for Ca_v_1.4 was clustered near elongated ribbons in cones of G369i KI mice (Fig. 1b). Thus, unlike in Ca_v_1.4 KO mice, the mutant Ca_v_1.4 protein is normally localized and supports the integrity of cone pedicles and ribbons in G369i KI mice.

**Figure 2.**
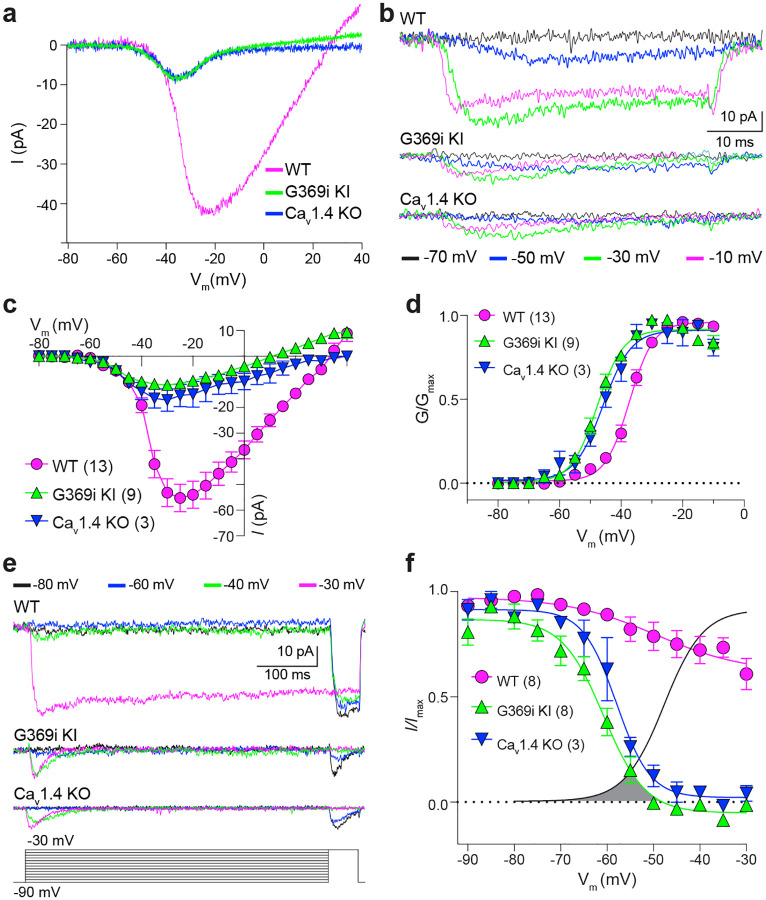
A low-voltage activated *I*_*Ca*_ is present in cones of G369i KI and Ca_V_1.4 KO but absent in WT mice. ***a,b***, Representative traces of *I*_*Ca*_ evoked by voltage ramps (a) and voltage steps (b). ***c,d***, I–V (c) and G–V (d) were plotted against test voltage for *I*_*Ca*_ evoked by 50-ms voltage steps from a holding voltage of −90 mV. Numbers of cells: WT, n = 13; G369i KI, n = 9; CaV1.4 KO, n = 3. ***e***, Representative *I*_*Ca*_ traces (top) and voltage-protocol (bottom) for steady-state inactivation. *I*_*Ca*_ was evoked by a conditioning pre-pulse from −90 mV to various voltages for 500 ms followed by a test pulse to −30 mV for 50 ms. ***f***, *I/I*_*max*_ represents the current amplitude of the test pulse normalized to current amplitude of the pre-pulse and was plotted against pre-pulse voltage. Numbers of cells: WT, n = 8; G369i KI, n = 8; Ca_v_1.4 KO, n = 3. In graphs in c,d, and f, smooth lines represent Boltzmann fits, symbols and bars represent mean ± SEM, respectively. In graph in f, line without symbols represents G-V curve for G369i KI cones replotted from d. Shaded region indicates window current for G369i KI cones.

We next compared patch clamp recordings of cones in retinal slices of adult WT, G369i KI, and Ca_v_1.4 KO mice under conditions designed to isolate *I*_*Ca*_ (Fig. 2). In WT cones, there was a large, sustained *I*_*Ca*_ that activated around −50 mV and peaked near −20 mV, consistent with the properties of Ca_v_1.4 (Fig. 2a–d). A small-amplitude *I*_*Ca*_ was detected in cones of G369i KI and Ca_v_1.4 KO mice but activated at significantly more negative voltages than in WT cones (Fig. 2a–d; Table 1). Moreover, *I*_*Ca*_ in the G369i KI and Ca_v_1.4 KO cones activated around −60 mV and peaked near −35 mV. This aberrant *I*_*Ca*_ was not sustained as in WT cones but inactivated rapidly during 50-ms (Fig. 2b) and 500-ms (Fig. 2e) step depolarizations. Overlay of the conductance-voltage (Fig. 2d) and inactivation curves (Fig. 2f) revealed a sizeable window current. These features of *I*_*Ca*_ in G369i KI and Ca_v_1.4 KO cones resembled those of Ca_v_3 T-type channels rather than Ca_v_1.4^20^.

**Figure 3.**
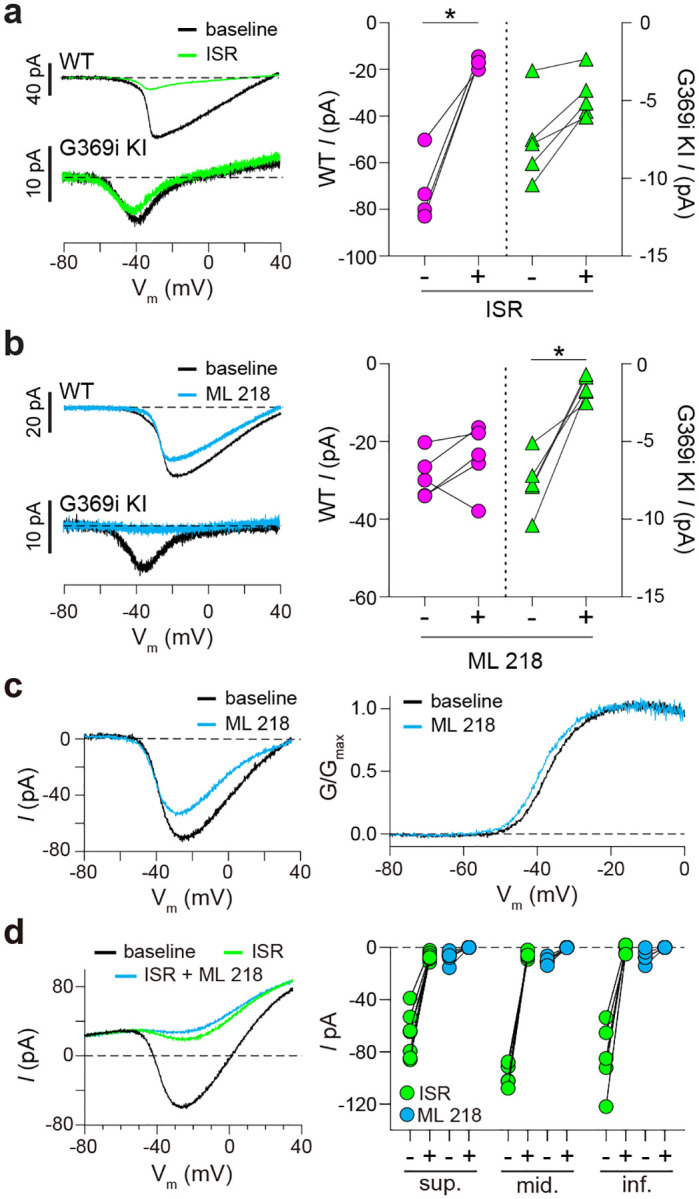
Pharmacological characterization of *I*_*Ca*_ in cones of WT and G369i KI mice and ground squirrel. ***a,b***, Analysis of mouse cones. *Left*, representative traces for *I*_*Ca*_ evoked by voltage ramps in cones of WT or G369i KI mice before (baseline) and after exposure to 1 μM of isradipine (ISR, *a*, WT, n = 4; G369i KI, n = 5) or 5 μM ML 218 (b, WT, n = 5; G369i KI, n = 5). *Right*, *I*_*Ca*_ amplitudes before (−) and during (+) perfusion of the blocker on the same cells. Each point represents a different cell. *, p < 0.05 by paired t-test. ***c***,***d***, Analysis of ground squirrel cones. Representative traces corresponding to baseline-corrected *I*_*Ca*_ evoked by voltage ramps (*c,d*, left) and corresponding G–V plot (*c*, right) before and during application of ML 218 (c) or ISR alone or ISR+ML218 (d). In *d, I*_*Ca*_ amplitudes are plotted before (−) and after (+) block for cones in the superior (sup.; n = 7 cones), middle (mid.; n = 5 cones), and inferior (inf.; n = 5 cones) thirds of the retina. The *I*_*Ca*_ blocked by ISR alone was measured at the peak of the control I–V curve between −40 and −20 mV. The *I*_*Ca*_ amplitude blocked by adding ML218 was measured as the average current between −50 and −45 mV before ML218 addition relative to zero current following the addition of ML218. The changes produced by ML218 were small but nonetheless significant (sup., n = 7, ISR: p < 0.0001, ML218: p = 0.0047; mid., n = 5, ISR: p < 0.0001, ML218: p = 0.0001; inf., n =5, ISR: p = 0.0019, ML218: p = 0.0460; two-tailed t test).

Although Ca_v_3 channels were reported in patch clamp recordings of cone pedicles in WT mouse cones^21^, we did not observe a low voltage-activated component of *I*_*Ca*_ in the I–V curve from our recordings of WT mouse cone somas, which would be indicative of a Ca_v_3 subtype (Fig. 2a,c). Moreover, *I*_*Ca*_ in WT cones was blunted by the Ca_v_1 antagonist isradipine (Fig. 3a) but not the Ca_v_3 antagonist, ML 218 (Fig. 3b; ML 218 caused a minor suppression of *I*_*Ca*_ in some WT cones, which could be attributed to weak activity on Ca_v_1.4, Supp.Fig. S1). In contrast, *I*_*Ca*_ in G369i KI cones was significantly suppressed by ML 218 but not by isradipine (Fig. 3a,b). To further test for a Ca_v_3 contribution to *I*_*Ca*_, we analyzed cones of ground squirrel retina, where the large amplitude of *I*_*Ca*_ facilitates pharmacological analyses. Consistent with its actions on Ca_v_1.4 in WT mouse cones (Fig. 3c) and transfected HEK293T cells (Supp.Fig. S1), ML 218 caused an insignificant inhibition of peak *I*_*Ca*_ (−7.0 ± 20.8%, n = 6 cones) as well as a negative shift in the voltage-dependence of activation in ground squirrel cones (Supp.Fig. S2). As a positive control, we confirmed that ML 218 blocked a prominent Ca_v_3-type current in ground squirrel type 3a OFF cone bipolar cells (Supp.Fig. S2). Application of isradipine followed by ML 218 resulted in a time-and voltage-dependent suppression of the residual *I*_*Ca*_, which is a hallmark of Ca_v_1 inhibition by dihyropyridine antagonists such as isradipine (Fig. 3d, Supp.Fig. S2)^22^. Finally, we also performed patch clamp recordings of cone terminals in macaque retina where there was no evidence of a Ca_v_3 current (Supp.Fig. S3). We conclude that Ca_v_3 channels contribute significantly to *I*_*Ca*_ in cones only when Ca_v_1.4 Ca^2+^ signals are absent.

### Cone synaptogenesis relies on the Ca_v_1.4 protein but not its Ca^2+^ conductance

**Figure 4.**
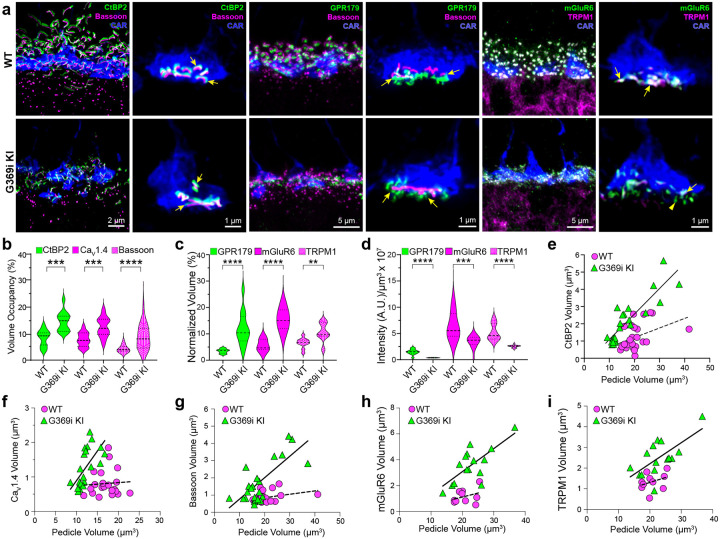
Immunofluorescence characterization of cone synapses in WT and G369i KI mice. ***a***, Confocal images of the OPL of WT and G369i KI mice labeled with antibodies against cone arrestin (CAR) and proteins that are presynaptic (CtBP2, bassoon) or postsynaptic (GPR179, TRPM1, mGluR6). Every other panel shows high-magnification, deconvolved images of single pedicles labeled with cone arrestin (rod spherule-associated signals were removed for clarity). Arrows indicate ribbon synapses, which appear enlarged in the G369i KI pedicles. ***b–d***, Violin plots represent volume occupancy of labeling for each synaptic protein normalized to their respective CAR-labeled pedicles. ***e–i***, Dependence of synapse size on pedicle size. Volumes corresponding to labeling of CtBP2 (*e*: p = 0.051, r = 0.4 for WT; p < 0.0001, r = 0.88 for G369i KI), Ca_V_1.4 (*f*: p = 0.8, r = 0.06 for WT; p = 0.002, r = 0.88 for G369i KI), bassoon (*g*: p = 0.1, r = 0.34 for WT; p < 0.0001, r = 0.75 for G369i KI), mGluR6 (*h*: p = 0.32, r = 0.35 for WT; p = 0.002, r = 0.73 for G369i KI) and TRPM1 (*i*: p = 0.32, r = 0.35 for WT; p = 0.007, r = 0.66 for G369i KI) are plotted against pedicle volume. Dashed and solid lines represent fits by linear regression for WT and G369i KI, respectively.

As shown in previous studies^7, 16–18^, CtBP2 labeled stubby, sphere-like structures resembling immature ribbon material in Ca_v_1.4 KO mice (Fig. 1). The presence of elongated ribbons in G369i KI mice suggested that, as in rods^19^, the non-conducting mutant Ca_v_1.4 protein may support the molecular assembly of cone synapses. To this end, we analyzed cone synapses by immunofluorescence and confocal microscopy. Along with the major constituents of the ribbon, CtBP2 and RIBEYE^23^, presynaptic proteins such as bassoon and members of the postsynaptic signaling complex in depolarizing (ON) cone bipolar cells (GPR179, mGluR6, and TRPM1^24^) were enriched near cone ribbons in G369i KI mice as in WT mice (Fig. 4a). Compared to WT mice, the labeled structures occupied a larger volume (Fig. 4b,c), albeit at a much lower density (Fig. 4d), which increased linearly with the volume of the pedicle in G369i KI mice (Fig. 4e–i), perhaps as a homeostatic response to Ca_v_1.4 loss-of-function.

**Figure 5.**
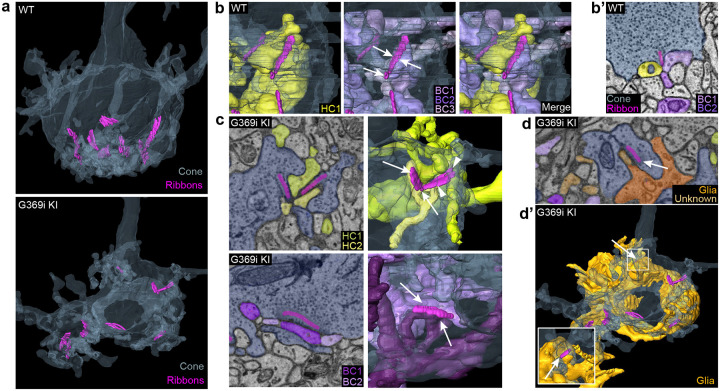
Cone synapses form incorrect pairings with postsynaptic partners in G369i KI mice. 3D reconstructions of WT and G369i KI pedicles (n = 2 each) were obtained by SBFSEM. ***a***, 3D renderings showing ribbons (magenta) within cone pedicles (gray) from WT and G369i KI mice. ***b,b’***, 3D renderings (b) show ribbon sites in a WT cone pedicle contacting one horizontal (HC1; yellow) and three bipolar cells (BC1–3; purple). The raw image (*b’*) shows a single plane example of BC1–2 and HC1 contacting the ribbon site. **c–d’**, Single plane raw images (left panels, *c; d*) and 3D reconstructions (right panels, *c*; *d’*) show ribbon sites within the G369i KI cone pedicle contacting in *c*: horizontal cells (HC1–2) only, CBCs only (BC1–2); and in *d,d’*: glia (the G369i KI cone pedicle contacts a glial cell (orange) and an unknown partner); the glial cell completely envelops the pedicle. Inset in *d’* shows glial-contacting ribbon site (arrow). In other panels, arrows indicate points of contact between ribbons and other postsynaptic elements.

The cone synapse is structurally complex, with the dendritic tips of two horizontal cells and an intervening ON cone bipolar cell invaginating deeply into the pedicle near the ribbon^3^. To test how the switch in Ca_v_ subtypes might affect this arrangement of postsynaptic partners, we generated 3D reconstructions of cone synapses by serial block-face scanning electron microscopy (SBFSEM; Fig. 5). As with the enlarged synaptic contacts (Fig. 4), there was some evidence of structural modifications in G369i KI pedicles. Compared to WT pedicles, ribbons appeared disorganized in G369i KI pedicles which extended telodendria laterally rather than apically (Fig. 5a). In addition, a slightly larger fraction of synaptic sites in the G369i KI pedicle (35% vs 14% in WT) formed incorrect postsynaptic partnerships (Fig. 5b–d’; Table 2). Nevertheless, the number of ribbons is normal in G369i KI cones and about half of the ribbons making invaginating contacts with the appropriate cell types (*i.e*., both horizontal cells and cone bipolar cells; Table 2). Therefore, while necessary for the structural refinement of the cone synapses, Ca_v_1.4 Ca^2+^ signals are largely dispensable for cone synapse assembly.

### Cone signaling to a postsynaptic partner is intact in G369i KI mice

**Figure 6.**
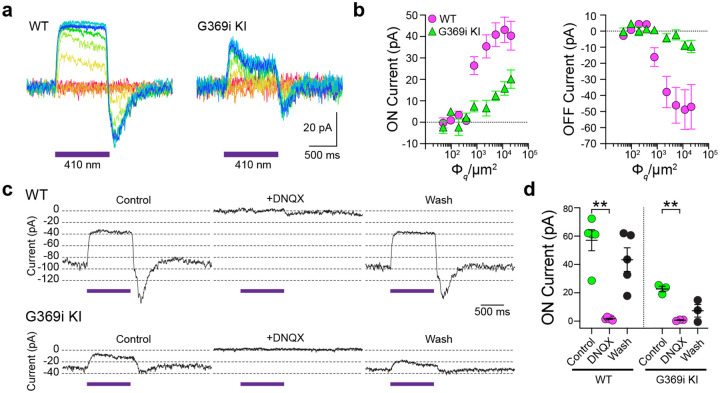
Light responses of horizontal cells are impaired in G369i KI mice. ***a-b***, Representative traces from whole-cell patch clamp recordings of horizontal cells held at −70 mV (*a*) and quantified data (*b*) for currents evoked by 1 s pulses of light (λ = 410 nm) plotted against light intensities. In *b*, peak current amplitudes during (ON current) and after (OFF current) the light stimuli were plotted against photon flux per μm^2^ (Φ_*q*_/μm^2^). Data represent mean ± SEM. WT, n = 8; G369i KI, n =9. ***c–d***, Representative traces from horizontal cells held at −70 mV (*c*) and quantified data (*d*) for currents evoked by 1-s pulses of light (λ = 410 nm, 1.2 × 10^5^ Φ_*q*_/μm^2^) before, during, and after washout of DNQX (20 μM). In *d*, symbols represent responses from individual cells, n = 5 cells for WT and 3 cells for G369i KI, bars represent mean ± SEM. **, p < 0.01 by paired t-tests.

The preservation of cone synapses in G369i KI mice allowed the unique opportunity to test whether a Ca_v_ subtype other than Ca_v_1.4 could support ribbon-mediated synaptic release. To this end, we compared synaptic transmission between cones and horizontal cells (HCs, Fig. 6) in retinal slices of WT and G369i KI mice. In darkness, glutamate released from cones depolarizes horizontal cells via activation of α-amino-3-hydroxy-5-methyl-4-isoxazolepropionic (AMPA)/kainate receptors^25, 26^. The resulting excitatory postsynaptic current (EPSC) undergoes a decline in response to light stimuli that hyperpolarize cones^27^. In WT HCs, a 1 s light pulse (λ=410 nm) inhibited the standing EPSC, which is reflected as an outward (hyperpolarizing “ON”) current (Fig. 6a). Upon termination of the light pulse, an inward (depolarizing “OFF”) current signaled the resumption of the EPSC (Fig. 6a). Both the ON and OFF components of the EPSCs in WT HCs increased with light intensity, reflecting the impact of luminance on the presynaptic membrane potential of cones and the subsequent change in glutamate release from their terminals (Fig. 6b). The ON and OFF components of EPSCs in G369i KI HCs were measurable, although lower in amplitude than in WT (Fig. 6a,b). The EPSC in darkness and the light response were abolished by the AMPA/kainate receptor antagonist, DNQX, in both WT and G369i KI horizontal cells (Fig. 6c,d). Thus, while greatly impaired, cone synapses can transmit light information to postsynaptic partners in the retina of G369i KI mice.

### Light responses of bipolar cells and visual behavior is spared in G369i KI but not Ca_v_1.4 KO mice

**Figure 7.**
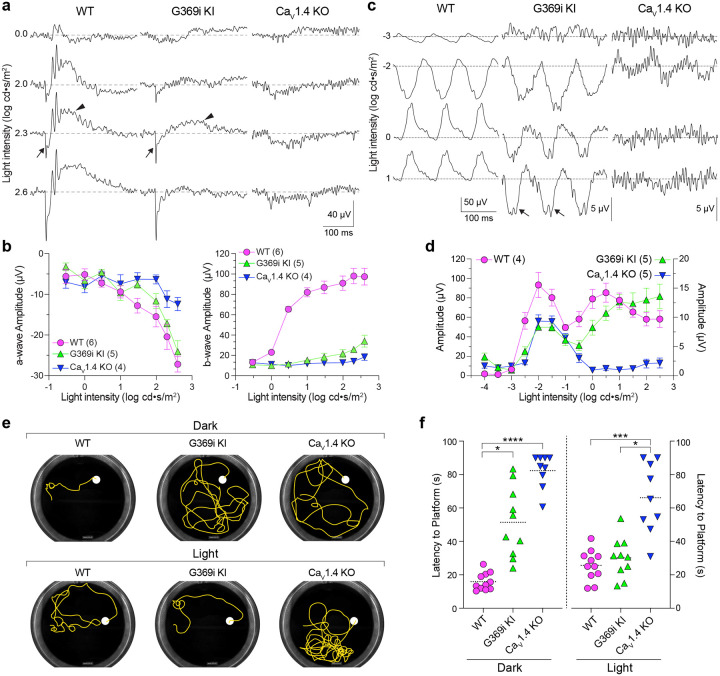
Photopic vision is reduced but not lost in G369i KI mice. ***a***, Representative traces of photopic ERGs recorded in the presence of background green light (20 cd · s/m^2^) in WT, G369i KI and Ca_V_1.4 KO mice. Flash intensities are shown at left. Arrows and arrowheads depict the a- and b-waves, respectively. ***b***, a-wave (left) and b-wave (right) amplitudes are plotted against light intensity. Symbols and bars represent mean ± SEM. WT, n = 7; G369i KI, n = 5; Ca_V_1.4 KO, n = 6. For a-waves, there was a significant effect of light intensity (p < 0.001) and genotype (p = 0.0086) by a mixed-effects model; by post-hoc Tukey test, there was no significant difference between WT and G369i KI at any light intensity. For b-waves at the highest light intensity, WT vs G369i KI, p = 0.0001; WT vs. Ca_V_1.4 KO, p < 0.0001; G369i KI vs. Ca_V_1.4 KO, p = 0.0038, Two-way ANOVA with Tukey’s post hoc analysis. ***c,d***, Representative traces (*c*) and quantified data (*d*) for 10 Hz flicker responses evoked by white light flashes of increasing luminance (from −4 to 2 log cd· s/m^2^). Arrows in *c* depict inverted waveform responses in G369i KI mice that are absent in CaV1.4 KO mice. Symbols and bars represent mean ± SEM. WT, n = 4; G369i KI, n = 5; Ca_V_1.4 KO, n = 5. At each of the 3 highest light intensities there was a significant difference (p < 0.05) in b-waves of WT vs G369i KI and G369i KI vs Ca_v_1.4 KO by Two-way ANOVA with Tukey’s post hoc analysis. ***e***, Representative swim path traces of WT, G369i KI and Ca_V_1.4 KO mice from the visible platform swim tests performed in the dark (upper traces) and light (lower traces). ***f***, Quantified latency to platform. Symbols represent the average of the last 3 swim trials for each mouse of each genotype for both dark and light conditions. Dotted lines represent the mean. WT, n = 11; G369i KI, n = 10; Ca_V_1.4 KO, n = 9. *, p < 0.05; ***, p < 0.001; ****, p < 0.0001; Kruskal-Wallis one-way ANOVA with Dunn’s post hoc analysis.

While horizontal cells act as inhibitory interneurons that modulate photoreceptor output, the vertical dissemination of visual information from cones to the inner retina is relayed by glutamate to ON and OFF cone bipolar cells (CBCs) which express mGluR6 and AMPA/kainate receptors, respectively. To test whether cone synaptic transmission to CBCs is intact in G369i KI mice, we recorded electroretinograms (ERGs) under light-adapted conditions using Ca_v_1.4 KO mice as a negative control (Fig. 7a–d). In these recordings, the light-induced response of photoreceptors and the postsynaptic response of ON CBCs corresponds to the a- and b-waves, respectively. Unlike in Ca_v_1.4 KO mice, the a-waves of G369i KI mice were like those in WT mice, which indicates that cones do not degenerate in this mouse strain. While reduced in amplitude, the b-wave was measurable in G369i KI mice and significantly larger than in Ca_v_1.4 KO mice at the highest light intensities (Fig. 7a,b). We also recorded flicker ERGs using 10 Hz light stimuli that can isolate cone pathways involving both ON and OFF CBCs. In WT mice, flicker responses exhibited two peaks, one at a lower irradiance (−2 log cd·s/m^2^) and one at higher irradiance (0.5 log cd·s/m^2^). The peak at the lower irradiance is attributed to responses in both rod and cone pathways, and the peak at higher irradiance is attributed to responses exclusively in cone pathways (Fig. 7c,d)^28, 29^. While significantly lower in amplitude than in WT mice, flicker responses in G369i KI mice showed a similar non-monotonic relation. Compared to Ca_v_1.4 KO mice, G369i KI mice showed peak flicker responses that did not differ at −2 log cd·s/m^2^, but were significantly higher at 0.5 log cd·s/m^2^ (Fig. 7d). The inverted flicker responses at higher illuminations in G369i KI mice (Fig. 7c) were absent in Ca_v_1.4 KO mice and may result from the hyperpolarizing contribution of cone-to-OFF CBC transmission. These results suggest that cone-to-CBC signaling is intact in G369i KI mice.

To validate these results with respect to vision-guided behavior, we used a swim test that assesses the ability of mice to identify a visible platform^30^. In darkness, G369i KI and Ca_v_1.4 KO mice took significantly longer to find the platform compared to WT mice (Fig. 7e,f). These results are consistent with our flicker response assays (Fig. 7c,d) as well as the absence of *I*_*Ca*_ in rods and rod-to-rod bipolar cell synaptic transmission in G369i KI mice^19^. In daylight conditions, G369i KI mice but not Ca_v_1.4 KO mice performed as well as WT mice (Fig. 7e,f). Thus, G369i KI mice are unique in retaining visual function under photopic but not scotopic conditions. Collectively, our results suggest that Ca_v_3 channels can support cone synaptic responses and visual behavior in G369i KI but not Ca_v_1.4 KO mice.

## DISCUSSION

The nervous system has remarkable abilities to adapt to pathological perturbations in neuronal activity. In the retina, ablation or degeneration of photoreceptors triggers various postsynaptic mechanisms that maintain some level of visual function in rod or cone pathways. These include remodeling of bipolar cell dendrites and their synapses^31–33^ as well as changes in the sensitivity of bipolar cells to photoreceptor input and inhibitory modulation^33, 34^. To our knowledge, this study provides the first evidence for a presynaptic form of homeostatic plasticity that originates within photoreceptors. Using Ca_v_1.4 KO and G369i KI mice, we identify the upregulation of a Ca_v_3 conductance as a common response to Ca_v_1.4 loss-of-function in cones. However, Ca_v_3 channels can only compensate for Ca_v_1.4 loss-of-function when cone synapse structure is maintained. Thus, our results also highlight a crucial, non-conducting role for the Ca_v_1.4 protein that allows cone synapses to function in the absence of Ca_v_1.4 Ca^2+^ signals.

### A non-canonical role for Ca_v_1.4 in regulating cone synapse assembly

A major finding of our study is that cone synapse formation requires the Ca_v_1.4 protein but not Ca_v_-mediated Ca^2+^ influx. As shown for rod synapses^19^, ribbons and other components of the pre- and post-synaptic complex assemble normally at cone synapses in G369i mice (Figs. 1,4,5). Ca_v_3 Ca^2+^ signals are dispensable for this process since their presence in Ca_v_1.4 KO cones is not accompanied by any semblance of ribbon synapses (Fig. 1B). An intimate relationship between Ca_v_1.4 and ribbons is supported by the colocalization of Ca_v_1.4 and RIBEYE puncta resembling ribbon precursor spheres in the developing OPL^7^. Moreover, light adaptation, which decreases the size of the ribbon, leads to a reduction in Ca_v_1.4 labeling in mouse retina^2^. While evidence for the binding of RIBEYE to Ca_v_1.4 is lacking, such an interaction could support the oligomerization of RIBEYE A and B domain within a macromolecular complex^35^. Alternatively, Ca_v_1.4 could pioneer sites of ribbon assembly perhaps by serving as a docking or nucleation site for the active zone. Regardless of the mechanism, our findings show that the formation of photoreceptor synaptic complexes does not require Ca^2+^ influx through Ca_v_1.4 channels.

Despite a normal molecular organization, G369i KI cone synapses were enlarged and made errors in postsynaptic partner selection. While I–V curves predict similar peak *I*_*Ca*_ amplitudes in cones of WT and G369i KI mice near the membrane potential of cones in darkness (−45 to −50 mV^36^, Fig. 1c), the strong inactivation of Ca_v_3 channels predicts greatly diminished Ca^2+^ signals in G369i KI pedicles. Paradoxically, reductions in presynaptic Ca^2+^ in rod photoreceptors are thought to cause illumination-dependent shrinkage of ribbons *in vivo* and *in vitro*^2, 37, 38^. However, an inverse correlation between presynaptic Ca^2+^ influx and ribbon synapse size is seen in sensory hair cells in zebrafish^39^. The mechanism involves decreased mitochondrial Ca^2+^ uptake and increase in the redox state of nicotinamide adenine dinucleotide (NAD^+^/NADH ratio)^40^. If a similar mechanism applied in cones, Ca_v_3 Ca^2+^ signals in G369i KI cones may decay too quickly to enable mitochondrial Ca^2+^ uptake mechanisms that trim ribbons. The size of ribbons and the postsynaptic specialization increased linearly with pedicle volume in G369i KI but not in WT mice (Fig. 4e–i), which could represent a form of homeostatic synaptic scaling as has been demonstrated at the neuromuscular junction^41, 42^. Similarly, the presence of some non-invaginating contacts with incorrect partner pairings in G369i KI mice (Fig. 5, Table 2) could represent a compensatory response to weakened synaptic output. Considering that only a subset of CBC subtypes re-wire correctly following partial ablation of cones in immature mice^32^, the structurally normal cone synapses of G369i KI mice could involve contacts with CBCs that are unusually resilient to loss of presynaptic input.

While it is well-established that Ca_v_1.4 is the main Ca_v_ subtype in mouse cones, Ca_v_3 channels, in particular Ca_v_3.2, have been reported to be expressed in mouse cones by electrophysiology^21^ and single cell RNA-seq studies^21, 43, 44^. By pharmacological and other criteria, we found no evidence for a functional contribution of Ca_v_3 in our recordings of cones in WT mice (Figs. 2,3), ground squirrels, or macaque (Supp.Figs. S2,S3). A caveat of using dihydropyridine antagonists such as isradipine to isolate the contribution of Ca_v_1 from other Ca_v_ subtypes is the relatively low sensitivity of Ca_v_1 channels to these drugs and their strong voltage-dependence. At a holding voltage of −90 mV, isradipine at 1 μM causes only ~80% inhibition of Ca_v_1.4 with greater block at depolarized voltages^22^, which agrees with our recordings of cones in WT mice (Fig. 3a) and ground squirrels (Fig. 3d, Supp.Fig.S2). Moreover, Ca_v_3 blockers such as Z944^45^ and ML218 (Supp.Fig. S2) have additional activity on Ca_v_1 channels at micromolar concentrations including effects on current amplitude and activation voltage. Thus, currents mediated by Ca_v_1.4 that are sensitive to Ca_v_3 blockers (e.g, >5 μM ML218) and spared by dihydropyridines at negative voltages may be mistaken as being mediated by Ca_v_3. In our experiments, the biophysical properties of the isradipine-sensitive *I*_*Ca*_ in cones of WT mice and ground squirrels resembled only those of Ca_v_1, whereas those of the ML218-sensitive *I*_*Ca*_ in cones of G369i KI mice resembled only those of Ca_v_3. Therefore, we favor the interpretation that Ca_v_3 contributes to *I*_*Ca*_ in mouse cones only upon silencing of the Ca_v_1.4 Ca^2+^ conductance.

Consistent with the diminutive Ca_v_3-mediated *I*_*Ca*_, light responses of HCs were evident but smaller in G369i KI than in WT mice. Due to their slow activation and strong inactivation^46^, Ca_v_3 channels may have a reduced ability to fuel the Ca^2+^ nanodomains that support fast and sustained components of release, both of which occur only at ribbon sites in cones^1 47^. Ca_v_3 channels may also be located further from the ribbon than Ca_v_1.4, thus lowering the efficiency of coupling to exocytosis. Unfortunately, it was not possible to test this with commercially available antibodies against Ca_v_3.2, which yielded identical patterns of immunofluorescence in the OPL of WT and Ca_v_3.2 KO mice (data not shown). A detailed analysis of the Ca_v_3 subtype(s) and their subcellular localization in G369i KI cones is required to unravel the shortcomings of Ca_v_3 channels with respect to cone synaptic release.

Based on extremely heterogeneous clinical presentations, CSNB2 manifests as a spectrum of visual disorders that originate from various mutations in *CACNA1F*^14, 48^. Even though some CSNB2 mutant Ca_v_1.4 channels may traffic normally to the plasma membrane, many of these mutations are expected to produce non-functional, non-conducting Ca_v_1.4 channels.^49, 50^ Yet, the visual phenotypes of CSNB2 patients are not as severe as the complete blindness in Ca_v_1.4 KO mice, which lack any Ca_v_1.4 protein expression and exhibit no signs of visual behavior (Fig. 7e,f)^51^. Collectively, our results suggest that G369i KI mice accurately model Ca_v_1.4 channelopathies in CSNB2 patients that are characterized by a greater impairment in rod than in cone pathways. This interpretation is supported by our findings that G369i KI mice exhibit horizontal cell responses to bright but not dim illumination (Fig. 6), ERG responses under conditions of light adaptation (Fig. 7a–d) but not dark-adaptation^19^, and visual behavior under photopic but not scotopic conditions (Fig. 7d). Together with the enlargement of synaptic sites, modest levels of synaptic release from cones of G369i KI mice may be sufficient to support nominal transmission of visual information through cone pathways. Furthermore, we acknowledge that G369i KI mice could also exhibit homeostatic alterations in the inner retina, which are known to support visual function when photoreceptor input is severely compromised^32–34^. Future studies of the retinal circuitry and visual behavior of G369i KI mice could identify compensatory pathways that are recruited upon Ca_v_1.4 loss-of-function and how they might be targeted in novel therapies for CSNB2 and related disorders.

## Methods

### Animals

All mouse and macaque experiments were performed in accordance with guidelines approved by the National Institutes of Health and the Institutional Animal Care and Use Committees at the University of Texas at Austin. All ground squirrel (*Ictidomys tridecemlineatus*) procedures performed at Northwestern University were approved by the Institutional Animal Care and Use Committee. The G369i KI^19^ and Ca_v_1.4 KO mouse strains were bred on the C57BL6/J background strain for at least 10 generations. Adult male and female mice were used (6–12 weeks old), and aged-matched C57BL6/J mice were used as the control (WT) animals.

### Immunofluorescence

Mice between 6–8 weeks of age were anesthetized using isoflurane and euthanized by cervical dislocation. Eyes were enucleated and hemisected. The eye cups with retina were fixed on ice in 4% paraformaldehyde in 0.1 M phosphate buffer (PB) for 30 min. Fixed eye cups were then washed three times with 0.1 M PB containing 1% glycine followed by infusion of 30% sucrose at 4°C overnight. The eye cups were orientated along their dorsal-ventral axis and frozen in a 1:1 (wt/vol) mixture of Optimal Cutting Temperature compound and 30% sucrose in a dry ice/isopentane bath. Eye cups were cryosectioned at 20 μm on a Leica CM1850 cryostat (Leica Microsystems), mounted on Superfrost plus Micro Slides (VWR), dried for 5 to 10 min at 42°C, and stored at −20°C until used. Slides with mounted cryosections were warmed to room temperature, washed with 0.1 M PB for 30 min to remove the OCT/sucrose mixture and blocked with dilution solution (DS, 0.1 M PB/10% goat serum/0.5% Triton-X100) for 15 min or overnight at room temperature. All remaining steps were carried out at room temperature. All primary antibodies and appropriate secondary antibodies were diluted in DS at concentrations specified in the Key Resources Table (Table 3). Sections were incubated with primary antibodies for 1 h or overnight and then washed five times with 0.1 M PB. Sections were then incubated with secondary antibodies for 30 min and then washed five times with 0.1 M PB. Trace 0.1 M PB was removed, and sections were then mounted with #1.5H coverslips (ThorLabs) using ProLong Glass Antifade Mountant with or without NucBlue (Thermo Fisher Scientific).

For double labeling with other rabbit polyclonal antibodies, CAR antibodies were conjugated with the CF647 fluorophore (CAR-647) using the Mix-n-Stain antibody labeling kit according to the manufacturer’s protocol (Biotium). Sections were processed first with rabbit polyclonal Ca_v_1.4 or EAAT2 antibodies and corresponding secondary antibodies as described above. To prevent CAR-647 from binding to any available sites on the anti-rabbit secondary antibodies previously added to the sections, Ca_V_1.4 or EAAT2 antibodies were readded to the sections and incubated for 30 min. After washing, the sections were incubated with CAR-647 for 1 h, followed by wash steps and cover glass mounting.

Immunofluorescence in labeled retinal sections was visualized using an Olympus FV3000 confocal microscope (Tokyo, Japan) equipped with an UPlanApo 60x oil HR objective (1.5 NA). Images were captured using the Olympus FLUOVIEW software package. Acquisition settings were optimized using a saturation mask to prevent signal saturation prior to collecting 16-bit. All confocal images presented are maximum z-projections. Images (256 × 256 pixels) used in analyses of synaptic proteins were collected using a 30X optical zoom, 0.6 Airy disk aperture, and voxel size of 0.028 *μm* × 0.028 *μm* × 0.2 *μm* (*X* × *Y* × *Z*). Amira segmentation was used for generating 3D binary masks, and Amira 3D label analysis was used for quantification of immunofluorescent and masked images. Non-deconvolved images were used for all analyses. 3D binary masks of individual CAR-labeled pedicles were made by setting the threshold 1 standard deviation above mean fluorescence intensity of each 3D image. 3D binary masks of presynaptic labels (CtBP2, Ca_V_1.4, and Bassoon) or postsynaptic labels (GPR179, mGluR6, and TRPM1) within or associated with the pedicle, respectively, were made by setting the threshold 3 standard deviations above the mean fluorescence intensity. To aid in presentation, high-mag images displayed in Figs. 3f, 4a, and 6f were deconvolved using cellSens software (Olympus), and any immunofluorescence corresponding to rod synaptic proteins was subtracted from the image.

### Molecular biology and transfection

The cDNAs for Ca_V_1.4 (GenBank: NM_019582), β_2X13_ (GenBank: KJ789960), and α_2_δ-4 (GenBank: NM_172364) were previously cloned into pcDNA3.1^52^. The cDNA for Ca_V_3.2 (GenBank: AF051946) was a gift from Dr. Edward Perez-Reyes, University of Virginia. All constructs were verified by DNA sequencing before use. Human embryonic kidney 293T (HEK293T) cells were cultured in Dulbecco’s Modified Eagle’s Medium with 10% FBS at 37°C in 5% CO_2_. At 70–80% confluence, the cells were co-transfected with cDNAs encoding human Ca_v_1.4 (1.8 μg) β_2X13_ (0.6 μg), α_2_δ-4 (0.6 μg), and enhanced GFP in pEGFP-C1 (Clonetech, 0.1 μg) or Ca_V_3.2 (2 μg) and pEGFP-C1 (0.1 μg) using FuGENE 6 transfection reagent according to the manufacturer’s protocol. Cells treated with the transfection mixture were incubated at 37°C for 24 hr, dissociated using Trypsin-EDTA, and replated at a low density to isolate single cells. Replated cells were then incubated at 30°C or 37°C for an additional 24 hr before beginning experiments.

### Solutions for patch clamp recordings

HEK293T extracellular recording solution contained the following (in mM): 140 Tris, 20 CaCl_2_, 1 MgCl_2_, pH 7.3 with methansulfonic acid, osmolarity 309 mOsm/kg. HEK293T internal recording solution contained the following: 140 NMDG, 10 HEPES, 2 MgCl_2_, 2 Mg-ATP, 5 EGTA, pH 7.3 with methansulfonic acid, osmolarity 358 mOsm/kg.

For recordings of *I*_*Ca*_ in mouse retina, extracellular recording solution contained the following (in mM): 115 NaCl, 2.5 KCl, 22.5 NaHCO_3_, 1.25 NaH_2_PO_4_, 2 CaCl_2_, 1 MgCl_2_, 5 HEPES, 5 CsCl, 5.5 Glucose, osmolarity 290 mOsm/kg. Mouse cone intracellular solution contained the following (in mM): 105 CsMeSO_4_, 20 TEA-Cl, 1 MgCl_2_, 11 HEPES, 10 EGTA, 4 Mg-ATP, 10 phosphocreatine, 0.3 Na-GTP, pH 7.4 with CsOH, osmolarity 300 mOsm/kg.

For recordings of *I*_*Ca*_ in ground squirrel retina, extracellular recording solution contained the following (in mM): 10 HEPES, 85 NaCl, 3.1 KCl, 2.48 MgSO_4_, 6 Glucose, 1 Na-succinate, 1 Na-malate, 1 Na-lactate, 1 Na-pyruvate, 2 CaCl_2_, 25 NaHCO_3_, and 20 TEA-Cl, osmolarity 285 ± 5 mOsm/kg. Intracellular solution contained the following (in mM): 80 CsCl, 10 BAPTA, 2 MgSO_4_, 10 HEPES, 20 TEA-Cl, 5 Mg-ATP, and 0.5 Na-GTP, pH 7.35 with CsOH, osmolarity 285 ± 5 mOsm/kg. For recordings of *I*_*Aglu*_, extracellular recording solution contained (in mM): 125 NaCl, 3.0 KCl, 1.25 NaH_2_PO_4_, 25 NaHCO_3_, 2 CaCl_2_, 1 MgCl_2_, 3 dextrose, 3 sodium pyruvate, 0.1 picrotoxin and 0.02 DNQX, and in indicated experiments 0.0013 TFB-TBOA. Intracellular I_Aglu_ contained (in mM): 125 KSCN, 10 TEA-Cl, 10 HEPES, 1 CaCl_2_, 2 MgCl_2_, 0.3 Na-GTP, 4 Mg-ATP, 10 K_2_ phosphocreatine, 0.02 ZD7288. Mouse and ground squirrel extracellular slice recording solutions were equilibrated with 5% CO_2_ / 95% O_2_ to a pH of ~7.5.

For recordings of light responses in horizontal cells of mouse retina, extracellular recording solution consisted of Ames’ media supplemented with 100 U/mL penicillin, 0.1 mg/mL streptomycin and 22.6 mM NaHCO_3_, osmolarity 280 ± 5 mOsm/kg. The intracellular recording solution contained the following (in mM): 135 K-Aspartate, 10 KCl, 10 HEPES, 5 EDTA, 0.5 CaCl_2_, 1 Mg-ATP, 0.2 Na-GTP, pH 7.35 with KOH, osmolarity 305 ± 5 mOsm/kg.

### Patch clamp electrophysiology

Whole-cell voltage clamp recordings of transfected HEK293T cells were performed 48 to 72 hrs after transfection using an EPC-10 amplifier and Patchmaster software (HEKA Elektronik, Lambrecht, Germany). Patch pipette electrodes with a tip resistance between 4 and 6 MΩ were pulled from thin-walled borosilicate glass capillaries (World Precision Instruments, Sarasota, FL) using a P-97 Flaming/Brown Puller (Sutter Instruments, Novato, CA). A reference Ag/AgCl wire was placed into the culture dish mounted on an inverted Olympus IX70 microscope. Recordings were performed at room temperature. A pressurized perfusion pencil multi-barrel manifold controlled with Valve Bank II (AutoMate Scientific, Inc., Berkeley, CA) was used to deliver extracellular solutions. ML218 of different concentrations (0.5, 1, 5, 25 and 100 mM) was added to the extracellular solution the day of experiments. Series resistance was compensated up to 70%, and passive membrane leak subtraction was conducted using a P/−4 protocol. Whole-cell Ca^2+^ currents (*I*_Ca_) of transfected HEK293T cells were evoked for 50 ms with incremental +5 mV steps from −80 mV to +65 mV. Current-Voltage (IV) data were fit with a single Boltzmann equation: *I*_*Ca*_ = *G*_*max*_*(V*_*m*_*–V*_*r*_)/(1+exp[-(*V*_*m*_-*V*_*h*_*))/k*]), where G_max_ is the maximal conductance, *V*_*m*_ is the test voltage, *V*_*r*_ is the Ca^2+^ reversal potential, *V*_*h*_ is the membrane potential required to activate 50% of *G*_*max*_, and k is the slope factor. Data were sampled at 100 kHz, filtered at 3 kHz, and analyzed using custom programs written in IgroPro (WaveMetrics).

Whole-cell voltage clamp recordings of mouse cones, macaque cone terminals, and mouse horizontal cells were performed using an EPC-10 amplifier and Patchmaster software (HEKA). Patch pipette electrodes with a tip resistance between 10 and 14 MΩ for cones and 6–8 for horizontal cells were pulled from thick-walled borosilicate glass (1.5 mm outer diameter; 0.84 mm inner diameter; World Precision Instruments).

To prepare retinal slices, adult mice (6 – 8 weeks old) were anesthetized using isoflurane and euthanized by cervical dislocation. Eyes were enucleated, placed into cold Ames’ media slicing solution, and hemisected. Following removal of the vitreous, the eye cup was separated into dorsal and ventral halves using a scalpel. Ventral retina was isolated, molded into low-melt agarose and mounted in a Leica VT1200s vibratome (Leica Biosystems). Mouse retina slicing solution was continuously bubbled with 100% O_2_ and contained the following: Ames’ Medium with L-glutamine supplemented with (in mM) 15 NaCl, 10 HEPES, 10 U/mL penicillin, 0.1 mg/mL streptomycin, pH 7.4, osmolarity 300 mOsm/kg. Vertical (~200 μm) or horizontal (~160 μm) retinal slices were anchored in a recording chamber, placed onto a fixed stage, and positioned under an upright Olympus BX51WI microscope equipped with a 60X water-immersion objective (1.0 NA), and superfused with extracellular solution (flow rate of ~1–2 ml/min) at room temperature. Slices were visualized using IR-DIC optics and an IR-2000 (Dage MTI, Michigan City, IN) or SciCam Pro (Scientifica, Uckfield, United Kingdom) CCD camera controlled by the IR-capture software package or μManager, respectively^53, 54^. Drugs used in these experiments were added to the mouse extracellular solution the day of experiments at the concentration described in Table 4. A reference Ag/AgCl pellet electrode was placed directly into the recording chamber solution. Data from whole-cell recordings with a series resistance >20 MΩ were discarded.

Cone somas were identified based on their morphology and location (outer ONL cell layer). Cone identity was confirmed by the whole-cell capacitance (~3–4 pF), which is larger than rod whole-cell capacitance (~0.7–1 pF). For cone voltage ramp recordings, cones were held at −90 mV for 200 ms followed by a ramp of +0.5 mV/ms to +40 mV. To determine voltage activation of *I*_Ca_, whole-cell Ca^2+^ currents in cones were evoked for 50 ms with incremental +5 mV steps from −80 mV to +40 mV. The activation voltage of *I*_Ca_ is reported as G/G_max_, where G is the conductance at each test voltage and G_max_ is the maximum peak conductance for each cone. Conductance was calculated using the equation *I*_*Ca*_ = G(V_m_–V_r_), where V_r_ is +60 mV. To determine steady state inactivation of *I*_Ca_, currents in cones were evoked for 500 ms with incremental +5 mV steps from −90 mV and −30 mV followed by a final step to −30 mV for 50 ms after each test voltage. The steady state inactivation of *I*_Ca_ is reported as *I/I*_*max*_, where *I* is the peak current in the final voltage step to −30 mV and *I*_*max*_ is the maximum peak current for each cone. Data were sampled between 20 and 60 kHz and filtered at 3 kHz.

For horizontal cell light responses, horizontal slices were prepared from central mouse retina. The identity of horizontal cells was determined based on their larger soma diameter (~15 μm) compared to bipolar cell somas (~6 μm). During whole cell patch clamp recordings, horizontal cells were held at −70 mV. Light stimuli (1 s) at 410 nm (630 × 830 μm) were presented onto the retina (at a minimum of 5 s intervals) through the microscope’s condenser using a Polygon1000 DMD pattern illuminator (Mightex, Pleasanton, CA, USA) and a custom-built light path. Light intensity (in watts) was measured at the point on the microscope stage where the retina is placed using a power meter (Thorlabs, Newton, New Jersey, USA). Photon flux Ф_*q*_ (photons/s) within the light stimulus area was calculated using the measured light intensities in the formula:

$$\phi_{q}\text{ per }\mu m^{2}=\frac{\text{measured light intensity}(W)}{\frac{hc}{\lambda }}/\text{light stimulus area}(\mu m^{2})$$

where *h* is Planck’s constant (J*s), *c* is the speed of light (m/s), and λ is the wavelength (m). Light stimulus intensity was increased in Log2 steps from 4.9×10^2^ to 2.1×10^5^ Ф_*q*_/μm^2^. For each light intensity step, both ON and OFF current amplitudes were measured from baseline (averaged 5 ms of current prior to light onset) to the maximum positive (after light onset) or maximum negative (after light offset) current, respectively. To confirm the identity of these horizontal cell light responses as AMPA-mediated currents, 1 s light stimuli (410 nm, 1.2×10^5^ Ф_*q*_/μm^2^) were continuously delivered every 10 s before, during and after bath application of 20 μM DNQX.

For voltage clamp recordings of 13-lined ground squirrel cones, retinal slices were prepared as previously described^55^. The eyecup was divided along the dorsal to ventral axis into superior, middle, and inferior parts. The dorsal area above the line of the optic nerve head was defined as superior, the central region with a width of about 5 mm just ventral to the optic nerve head was middle, and the remaining ventral area was inferior. Isradipine and ML218, alone or in combination, were applied from separate puffer pipettes whose orifices were aimed at the cone synaptic region. Recordings were made with an Axopatch 200B amplifier (Molecular Devices). Signals were electronically filtered at 5 kHz and digitized at a rate of 10 kHz. Additional Gaussian filtering was added (cutoff frequency of 500 Hz). Tissue was viewed through a 63x water immersion objective on a Zeiss Axioskop FS2 microscope and superfused with extracellular solution at room temperature. Drugs and their concentrations used during these experiments are described in Table 4. Membrane potential was continuously maintained at −85 mV. During ramp stimulation, the membrane potential was depolarized to −85 to +35 mV at a rate of 1 mV/ms.

For rhesus macaque cone terminal recordings, a 12-year-old male was sedated with ketamine (5 mg/kg I.M.) and dexmedetomidine (0.015 mg/kg I.M.). Post-sedation, the animal received buprenorphine (0.02 mg/kg I.M.), atropine (0.02 mg/kg I.M.), and maropitant citrate (1 mg/kg S.Q.). The animal was intubated and maintained with inhaled isoflurane (0.75–2.0%) and propofol (7–8 mg/kg/hr I.V.). Crystalloid fluids (5 mL/kg/hr) were administered I.V., and phenylephrine (5–10 mcg/kg/hr I.V.) was used for blood pressure support. The animal was under anesthesia for approximately 3 hours prior to perfusion. Transcardial perfusion was approached through a midline thoracotomy. Immediately prior to perfusion, 5 mL of Euthasol^®^ (sodium pentobarbital 390 mg/mL/phenytoin sodium 50 mg/mL) was administered I.V. The descending thoracic aorta was clamped, the pericardium was opened, and the right atrium was cut. The apex of the left ventricle was sharply incised, and a large bore cannula (Yankauer suction handle, 5 mm internal diameter) was inserted through the left ventricle until it could be palpated in the ascending aorta. The cannula was clamped in place at the apex of the left ventricle. The animal was perfused with 4000 mL of cold phosphate-buffered saline at ~500 mL/min. Eyes were removed approximately 1 hour following perfusion. Eyes were dissected, and eye cups were allowed to dark adapt for ~30 min and stored in bicarbonate buffered Ames’ media at 32°C equilibrated with 95% O_2_/5% CO_2_ prior to slice preparations. Vertical sections of central retina were prepared from 5 mm retina punches as described for mice.

### Serial block-face scanning electron microscopy and 3D reconstructions

Eye cups were prepared from P42 WT and G369i KI littermates and fixed using 4% glutaraldehyde in 0.1M cacodylate buffer, pH 7.4, for 4 hours at room temperature followed by additional fixation overnight at 4°C. Glutaraldehyde-fixed eye cups were then washed 3 times in 0.1M cacodylate buffer. Retinas were thereafter isolated and embedded in Durcupan resin after staining, dehydration, and embedding as described previously (Della Santina et al., 2016). A Thermo Scientific VolumeScope serial block face scanning electron micrscope was used to image embedded retinas. Retinal regions comprising a 2×2 montage of 40.96 μm tiles were imaged at a resolution of 5nm/pixel and section thickness of 50 nm. Image stacks were aligned, and cone photoreceptor terminals reconstructed using TrakEM2 (NIH). Postsynaptic partners at cone ribbons were followed to the inner nuclear layer to determine their identity. Amira software was used for 3D visualization of reconstructed profiles.

### Electroretinography

Retinal function was assessed using the Celeris system (Diagnosys, Inc.) paired with the Espion software (Diagnosys, Inc.). Mice were anaesthetized under red light (660 nm) via intraperitoneal (I.P.) injection with ketamine/xylazine mixture (100 mg/kg ketamine, 10 mg/kg xylazine). Tropicamide ophthalmic solution (1 %) and hypromellose lubricant eye gel (0.3 %) were administered topically to both eyes before the mouse was secured to a heated (37 °C) platform to maintain body temperature. Ag/AgCl corneal stimulators were placed on each eye. After collecting data from individual mice, atipamezole (Antisedan, 1–2 mg/kg) was I.P. injection administered to reverse the effects of the ketamine and xylazine. For photopic ERGs, eyes were light adapted using a background green light at 20 cd·s/m^2^ for 10 min. Following light adaptation, eight different light intensity pulses (−0.5, 0, 0.5, 1, 1.5, 2, 2.3 and 2.6 log cd·s/m^2^) were delivered on top of the background green light. ERG a-waves were measured from baseline to the peak of the negative potential. ERG b-waves were measured from the peak of the a-wave to the peak of the positive potential. For flickering ERGs, mice were dark adapted (>12 hrs). White light pulses from −4 to 2.5 log cd·s/m^2^ were delivered in 0.5 log unit steps at 10 Hz. Response amplitudes were measured from the trough to the peak of each response at all light intensities. Each intensity stimulus was delivered 10 times, with a 3 s interval between each stimulus, and averaged. The mean response amplitudes recorded in the right and left eye of each mouse are reported for all quantified ERG data.

### Visible platform swim test

The visible platform swim test was performed as has been previously described^30^. WT, G369i KI, and Ca_v_1.4 KO male and female mice (6–9 weeks old) were adapted to the procedure room for at least 1 h prior to beginning the experiments. A water-filled, 4-foot diameter galvanized steel tank and a visible white platform with a diameter of 10 cm was used for the swim test. Mice were subjected to 6 swim trials per day. Assays conducted under photopic (55 lux) and scotopic conditions (0 lux) were performed on days 1 and 2, respectively. Light intensity for photopic and scotopic conditions was measured at the platform using an Extech HD450 light meter (FLIR Systems, Nashua, New Hampshire). Mice were given 90 s to find the platform before being removed. After one trial was performed on all mice, the platform was moved to one of three different locations, top left, top center, and top right in relation to the initial site of mouse placement in tank. Each trial was recorded using an infrared camera (Basler AG, Ahrensburg, Germany) and EthoVision XT16 software (Noldus Information Technology). Locating the platform was considered successful when mice contacted the platform with a head-on approach, even if mice failed to escape onto the platform. Mice were allowed to rest on the platform for 15 s at the end of each trial before being returned to a pre-warmed cage. Latency to find the platform was manually recorded and confirmed using recorded videos. Male and female mice were tested separately, and no sex differences in performance were identified post-hoc using two-way RM-ANOVA (Supplementary Table 1). Male and female data were combined into a single group for each genotype. Latency to platform was calculated by averaging the final 3 trials under scotopic and photopic conditions for each mouse and compared using Kruskal-Wallis one-way ANOVA with Dunn’s posthoc multiple comparison test.

### Data analysis

Electrophysiological data were analyzed by custom routines written in IgorPro software (Wavemetrics) and statistical analysis was performed using Prism software (GraphPad). Data were analyzed for normality by Shapiro Wilk test followed by parametric (t-test) or non-parametric methods (Kruskal Wallis or Mann-Whitney).

## Supplementary Material

1

## Figures and Tables

**Table 1.** Comparison of parameters from electrophysiological recordings of cones.

	C_M_ (pF)	P-value	n	R_M_ (MΩ)	P-value	n
WT	4.88 (3.86, 5.01)	--	11	3.57 (2.81, 4.65)	--	11
G369i KI	4.08 (3.88, 4.34)	0.73^a^	9	5.06 (4.04, 8.24)	0.04^a^	9
Ca_v_1.4 KO	3.31 (3.00, 3.76)	0.001^a^, 0.04^b^	6	8.21 (7.26, 9.06)	0.001^a^, 0.47^b^	6
	
G-V	*V*_*h*_ (mV)	P-value	n	*k*	P-value	n
WT	−37.54 (−38.91, −35.13	--	13	2.75 (2.47, 4.32)		13
G369i KI	−47.59 (−50.07, −46.72)	0.0001^a^	9	2.99 (2.68, 4.16)	0.99^a^	9
Ca_v_1.4 KO	−44.98 (−50.15, −43.27)	0.085^a^, 0.99^b^	3	4.33 (3.73, 5.22)	0.34^a^, 0.49^b^	3
	
Steady state inactivation	*V*_*h*_ (mV)	P-value	n	*k*	P-value	n
WT	−49.06 (−57.95, −41.82)	--	8	−7.25 (−12.30, −4.97)	--	8
G369i KI	−59.40 (−62.73, −58.01)	0.01^a^	8	−3.81 (−5.23, −2.46)	0.12^a^	8
Ca_v_1.4 KO	−56.62 (−61.65, −56.56)	0.88^a^, 0.88^b^	3	−3.67 (−5.17, −1.19)	0.14^a^, 0.99^b^	3
	
	Tau activation	P-value	n	Tau deactivation	P-value	n
WT	2.04 (1.26, 2.71)	--	11	0.88 (0.62. 2.09)	--	6
G369i KI	3.32 (2.99, 4.06)	0.001^a^	9	3.40 (2.62, 4.34)	0.004^a^	6

Values represent median (25^th^, 75^th^ quartiles). *V*_*h*_ and *k* were determined from Boltzmann fits of the G-V and steady-state inactivation curves. Time constant (tau) for activation was obtained from exponential fit of the rising phase of *I*_*Ca*_ evoked by a 50-ms test pulse to a voltage near the peak of the I–V. Tau deactivation was determined from exponential fit of the decay of the tail current evoked by repolarization to −90 mV from +20 mV. C_M_, membrane capacitance; R_M_, input resistance. P-values were determined by Kruskal Wallis test.

a, relative to WT.

b, relative to G369i KI.

**Table 2.** Comparison of parameters for cone synapse organization

	WT		G369i KI	
	Cone 1	Cone 2	Cone 1	Cone 2
# of ribbons	12	8	8	6
				
Type of contact: % total (fraction of total)				
*Invaginating*	58.3 (7/12)	87.5 (7/8)	50 (4/8)	50 (3/6)
*Non-invaginating*	41.7 (5/12)	12.5 (1/8)	50 (4/8)	50 (3/6)
				
Partner composition: % total (fraction of total)				
*HC/BC*	83.3	87.5	50.0	66.7
*HC only*	8.3	12.5	25.0	33.3
*BC only*	8.3	0	12.5	0
*Glia only*	0	0	12.5	0

Results represent analysis of n = 2 pedicles reconstructed by serial block face scanning electron microscopy in images obtained from WT or G369i KI mice (N= 1 animal each). Sites were counted as ‘invaginating’ if postsynaptic partners were enveloped on all sides by the cone terminal for at least a couple of consecutive image planes.

**Table 3.** Key resources used in this study.

*Primary Antibodies*				
Antibody Name (clone/designation)	Working Concentration	Source	Identifier	RRID
Bassoon (SAP7F407/Ms monoclonal)	2 μg/mL	ThermoFisher Scientific	MA1–20689	AB_2066981
Ca_V_1.4 (Rb Polyclonal)	5 μg/mL	Dr. Amy Lee (Lui et al., 2013)	Ab167	AB_2650487
Cone Arrestin (Rb Polyclonal)	1 μg/mL	Millipore	AB15282	AB_1163387
Cone Arrestin-CF647 (Rb Polyclonal)	5 μg/mL	Millipore	AB15282	AB_1163387
CtBP2 (Clone 16/Ms Monoclonal)	1 μg/mL	BD Biosciences	612044	AB_399431
GPR179 (mAB 1a/Ms Monoclonal)	2 μg/mL	Millipore	MAB427	AB_2069582
mGluR6 (366/Ms Monoclonal)	1 μg/mL	Dr. Theodore Wensel (Agosto et al., 2014, 2018)	N/A	N/A
TRPM1 (545H5/Ms Monoclonal)	1 μg/mL	Dr. Theodore Wensel (Agosto et al., 2018)	N/A	N/A
*Secondary Antibodies*				
Antibody Name and Conjugate	Working Concentration	Source	Identifier	RRID
Goat Anti-Mouse IgG1 Alexa Fluor 488	4 μg/mL	ThermoFisher	A-21121	AB_2535764
Goat Anti-Mouse IgG2a Alexa Fluor 555	4 μg/mL	ThermoFisher	A-21137	AB_2535776
Goat Anti-Rabbit Alexa Fluor 555	4 μg/mL	ThermoFisher	A-21429	AB_2535850
Goat Anti-Rabbit Alexa Fluor 647	4 μg/mL	ThermoFisher	A-21245	AB_2535813

*Chemicals*		
Reagent or Resource	Source	Identifier
Adenosine 5’-triphosphate magnesium salt	SIGMA	A9187
Agarose, low gelling temperature	Sigma-Aldrich	A0701
AMES	Sigma, US Biological	Sigma: A1420; US Biological: A1372
Antisedan	Zoetis US LLC	10000449
1,2-Bis(2-aminophenoxy)ethane-N,N,N’,N’-tetraacetic acid (BAPTA)	Sigma-Aldrich	A4926
Cadmium Chloride	Sigma	202908
Calcium chloride dihydrate	Sigma-Aldrich	223506
Cesium Chloride	Fisher Bioreagents	BP210
Cesium methanesulfonate	Sigma-Aldrich	C1426
Creatine phosphate, dipotassium salt	EMD Millipore	237911
D-Glucose	Fisher	D16
DL-*threo*-β-Benzyloxyaspartic acid (DL-TBOA)	Tocris	1223
6,7-Dinitroquinoxaline-2,3-dione disodium salt (DNQX)	Abcam	ab120169
Dimethyl sulfoxide	SIGMA	D2650
Dulbecco’s Modified Eagle Medium, high glucose	Gibco	11965–092
Dulbecco’s Phosphate Buffered Saline, no calcium, no magnesium	Gibco	14190–144
Ethylene glycol-bis(2-aminoethylether)-N,N,N’,N’-tetraacetic acid (EGTA)	Sigma-Aldrich	03777
Fetal Bovine Serum (FBS)	Gibco	16140–071
FuGENE HD Transfection reagent	Promega	E2311
Guanosine 5’-triphosphate sodium salt hydrate	Sigma-Aldrich	G8877
4-(2-Hydroxyethyl)piperazine-1-ethanesulfonic acid (HEPES)	SIGMA	H3375
Systane Hypromellose lubricant eye gel	Alcone	300650474016
Isradipine	Sigma-Aldrich	I6658
Ketamine Injection C IIIN 100 mg/mL	Dechra Veterinary Products, LLC	17033-100-10
Magnesium Chloride hexahydrate	SIGMA	M2393
Methanesulfonic acid	Sigma-Aldrich	471356
Mix-n-Stain Antibody Labeling Kit	Biotium	92238
ML218 Hydrochloride	Tocris	4507
*N*-Methyl-D-glucamine (NMDG)	Sigma-Aldrich	66930
Nickel Chloride	Sigma	339350
Optimal Cutting Temperature compound	Sakura	4583
Penicillin-Streptomycin	SIGMA	P0781
Picrotoxin	Tocris	1128
Sodium Bicarbonate	MACRON	7412–12
Sodium phosphate monobasic anhydrous	Sigma	S3139
Sodium phosphate dibasic anhydrous	Sigma	RDD022
Sodium Chloride, Crystal	MACRON	7581–12
Potassium Chloride	Fisher Chemical	P217
Sodium hydroxide	Fisher chemical	S318
Sodium lactate	Sigma-Aldrich	1614308
Sodium malate	Sigma-Aldrich	1613881
Sodium pyruvate	Sigma-Aldrich	P2256
Sodium succinate	Sigma-Aldrich	S9637
Strychnine HCl	Sigma	S8753
(3S)-3-[[3-[[4-(Trifluoromethyl)benzoyl]amino]phenyl]methoxy]-L-aspartic acid (TBF-TBOA)	Tocris	2532
Tetraethylammonium chloride (TEA-Cl)	Sigma-Aldrich	86614
TRIS	VWR	0497
Tropicamide ophthalmic solution 1%	Akorn, Inc.	17478-0102-12
Trypsin-EDTA (0.05%), phenol red	Gibco	25300054
Anased (Xylazine) Injection 100 mg/mL	Pivetal	46066-0750-02
4-Ethylphenylamino-1,2-dimethyl-6-methylaminopyrimidinium chloride (ZD7288)	Tocris	1000

*Experimental Models: Organisms/Strains*			
Reagent or Resource	Source	Identifier	RRID
HEK293T	ATCC	CRL-3216	CVCL_0063
Mouse: C57BL/6J	Jackson Labs	000664	IMSR_JAX:000664
Mouse: G369i KI	A. Lee^19^	N/A	N/A
Mouse: Cav1.4 KO	Jackson Labs	017761	IMSR_JAX:017761
Ground Squirrel	TLC	--	--

*Software and Algorithms*		
Reagent or Resource	Source	Identifier
Amira	ThermoFisher Scientific	https://www.thermofisher.com/amira
cellSens	Olympus	https://www.olympus-lifescience.com/en/software/cellsens
Espion software	Diagnosys, Inc.	https://info.diagnosysllc.com/software
FLUOVIEW	Olympus	https://www.olympus-lifescience.com/en/laser-scanning/fv3000
GraphPad Prism	GraphPad	https://www.graphpad.com
IGOR Pro	WaveMetrics	https://www.wavemetrics.com
ImageJ	NIH	https://imagej.nih.gov/ij
pClamp	Molecular Devices	https://www.moleculardevices.com
Patchmaster	Harvard Bioscience, Inc.	https://www.heka.com

**Table 4.** Pharmacological drugs and their concentrations used in electrophysiological recordings of cones.

Compound	Concentration (μM) for mouse recordings	Concentration (μM) for ground squirrel recordings
Cadmium	200	N/A
DL-TBOA	N/A	375
DNQX	20	N/A
Isradipine	1	2
ML218	5	5
Nickel	100	N/A
Picrotoxin	N/A	50
Strychnine	N/A	10
ZD7288	N/A	50
Glutamate	1000	N/A
