# Supplementary material for: A non-conducting role of the Cav1.4 Ca2+ channel drives homeostatic plasticity at the cone photoreceptor synapse
Source: bioRxiv. 2024 Aug 6:2023.12.05.570129. Originally published 2023 Dec 6. Preprint. [Version 3] doi: 10.1101/2023.12.05.570129 (PMC10723350; doi:10.1101/2023.12.05.570129)
Supplement: Supplement 1 [file NIHPP2023.12.05.570129v3-supplement-1.pdf]

Supplementary Figures

Figure 2- Figure Supplement 1. Voltage ramps do not reveal *I_{Ca}* in rods of G369i KI mice. ***a,b***, Representative traces of *I_{Ca}* evoked by voltage ramps at 0.15 mV/ms (left) or 0.5 mV/ms (right) in rods of WT or G369i KI mice. ***c***, Comparison of current amplitudes evoked by the different voltage ramps in rods of WT and G369i KI mice. Bars represent mean ± SEM. Each point represents a different cell. p-values were determined by unpaired t-test (t(12)=7.428 for 0.15 mV/ms, t(10) = 6.028 for 0.5 mV/ms).

Figure 3- Figure Supplement 2. Effect of ML218 on $Ca_v1.4$ and $Ca_v3.2$ in HEK293T cells. Cells were transfected with $Ca_v1.4$, β_{2x13}, and $\alpha_2\delta{-}4$ (a-d) or $Ca_v3.2$ (e-h). ***a,e,*** Representative

bioRxiv preprint doi: https://doi.org/10.1101/2023.12.05.570129; this version posted August 6, 2024. The copyright holder for this preprint (which was not certified by peer review) is the author/funder, who has granted bioRxiv a license to display the preprint in perpetuity. It is made available under aCC-BY 4.0 International license.

traces and I-V plots for I_{Ca} evoked by 50 ms steps to +10 mV (a) or 200 ms steps to -35 mV (e) from a holding voltage of -90 mV. Currents were recorded in the presence of vehicle (DMSO) or ML 218 (5 µM for $Ca_v1.4$ and 1 µM for $Ca_v3.2$). ***b,f,*** Left, I-V relationships for data obtained as in *a,e* but using steps to various voltages in a single representative cell. *Right*, Boltzmann fit of the responses, normalized to the maximum I_{Ca} amplitude. ***c,d,g,h***, Graphs depicting effect of ML 218 on V_{half} (c,g) and I_{Ca} amplitude (d,h) ** W=-93.00, Z=-3.1366, p=0.0017; *** W=105.0, Z=3.8419, p=0.0001; both by Wilcoxon matched pairs signed rank test. * t(2)=0.582, p=0.0366 by paired t-test.

Figure 2- Figure Supplement 3. Violin plots showing expression levels of transcripts at the single cell level in the retina of WT and G369i KI mice. Each point represents result from a single cell. a, Expression levels of transcripts known to be expressed in cones. Cells were grouped according to their transcriptional profiles using unsupervised clustering. Clusters 8 and 16 were identified as cones based on their high expression of Arr3, Opn1mw, Opn1sw, and Gnat2. b, Expression of transcripts that encode Cav1.4 (Cacna1f) and Cav3 subtypes (Cacna1g, Cacna1h, Cacna1i) in clusters 8 and 16 in retina from WT and G369i KI mice. In cluster 8, there was a small but significant decrease in the level of Cacna1f in the G369i KI vs WT (*, p = 0.01) but not in cluster 16 (p =0.09). There was no significant difference in the level of Cacna1h between genotypes in cluster 8 or 16 (p = 1).
