# Supplementary material for: A non-conducting role of the Cav1.4 Ca2+ channel drives homeostatic plasticity at the cone photoreceptor synapse
Source: bioRxiv. 2024 Jun 17:2023.12.05.570129. Originally published 2023 Dec 6. Preprint. [Version 2] doi: 10.1101/2023.12.05.570129 (PMC10723350; doi:10.1101/2023.12.05.570129)
Supplement: Supplementary file 1 [file NIHPP2023.12.05.570129v2-supplement-1.pdf]

bioRxiv preprint doi: https://doi.org/10.1101/2023.12.05.570129; this version posted June 17, 2024. The copyright holder for this preprint (which was not certified by peer review) is the author/funder, who has granted bioRxiv a license to display the preprint in perpetuity. It is made available under aCC-BY 4.0 International license.

Supplementary Table 1. Statistical analysis of visible platform swim test disaggregated by sex

**Male versus Female WT (Photopic)**	SS	DF	MS	F (DFn, DFd)	P value
Trial x Genotype	1125	5	224.9	F (5, 45) = 0.6060	P=0.6956
Trial	3564	5	712.9	F (5, 45) = 1.921	P=0.1096
Genotype	273.6	1	273.6	F (1, 9) = 0.6616	P=0.4370
Subject	3722	9	413.5	F (9, 45) = 1.114	P=0.3727
Residual	16703	45	371.2		
**Male versus Female G369i KI (Photopic)**	SS	DF	MS	F (DFn, DFd)	P value
Trial x Genotype	1457	5	291.4	F (5, 45) = 0.5362	P=0.7477
Trial	3516	5	703.2	F (5, 45) = 1.294	P=0.2833
Genotype	1150	1	1150	F (1, 9) = 0.8168	P=0.3897
Subject	12675	9	1408	F (9, 45) = 2.592	P=0.0168
Residual	24451	45	543.4		
**Male versus Female Cav1.4 KO (Photopic)**	SS	DF	MS	F (DFn, DFd)	P value
Trial x Genotype	1736	5	347.1	F (5, 35) = 0.6597	P=0.6563
Trial	993	5	198.6	F (3.056, 21.39) = 0.3775	P=0.7737
Genotype	78.37	1	78.37	F (1, 7) = 0.03788	P=0.8512
Subject	14481	7	2069	F (7, 35) = 3.932	P=0.0029
Residual	18416	35	526.2		
**Male versus Female WT (Scotopic)**	SS	DF	MS	F (DFn, DFd)	P value
Trial x Genotype	862	5	172.4	F (5, 45) = 0.6907	P=0.6331
Trial	914.5	5	182.9	F (5, 45) = 0.7328	P=0.6027
Genotype	634.7	1	634.7	F (1, 9) = 2.121	P=0.1793
Subject	2693	9	299.3	F (9, 45) = 1.199	P=0.3193
Residual	11232	45	249.6		
**Male versus Female G369i KI (Scotopic)**	SS	DF	MS	F (DFn, DFd)	P value
Trial x Genotype	7648	5	1530	F (5, 45) = 2.581	P=0.0390
Trial	1644	5	328.8	F (5, 45) = 0.5547	P=0.7339
Genotype	4712	1	4712	F (1, 9) = 4.716	P=0.0580
Subject	8993	9	999.2	F (9, 45) = 1.686	P=0.1206
Residual	26671	45	592.7		
**Male versus Female Cav1.4 KO (Scotopic)**	SS	DF	MS	F (DFn, DFd)	P value
Trial x Genotype	1727	5	345.4	F (5, 35) = 1.470	P=0.2246
Trial	12215	5	2443	F (2.802, 19.62) = 10.40	P=0.0003
Genotype	96.33	1	96.33	F (1, 7) = 0.1346	P=0.7246
Subject	5011	7	715.9	F (7, 35) = 3.046	P=0.0131
Residual	8225	35	235		

Data were analyzed by two-way repeated measures ANOVA. SS, sums of squares; DF, degrees of freedom; MS, mean square; F(DFn, DFd), F statistic.

bioRxiv preprint doi: https://doi.org/10.1101/2023.12.05.570129; this version posted June 17, 2024. The copyright holder for this preprint (which was not certified by peer review) is the author/funder, who has granted bioRxiv a license to display the preprint in perpetuity. It is made available under aCC-BY 4.0 International license.

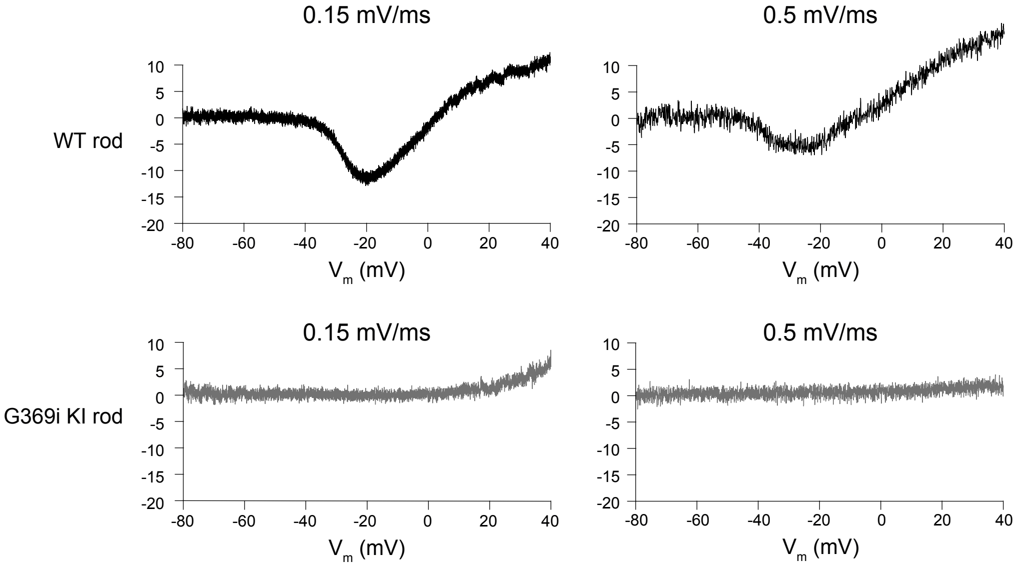

Supplementary Figure S1. Voltage ramps do not reveal I_{Ca} in rods of G369i KI mice. I_{Ca} was evoked by voltage ramps at 0.15 mV/ms or 0.5 mV/ms in rods of WT or G369i KI mice.

bioRxiv preprint doi: https://doi.org/10.1101/2023.12.05.570129; this version posted June 17, 2024. The copyright holder for this preprint (which was not certified by peer review) is the author/funder, who has granted bioRxiv a license to display the preprint in perpetuity. It is made available under aCC-BY 4.0 International license.

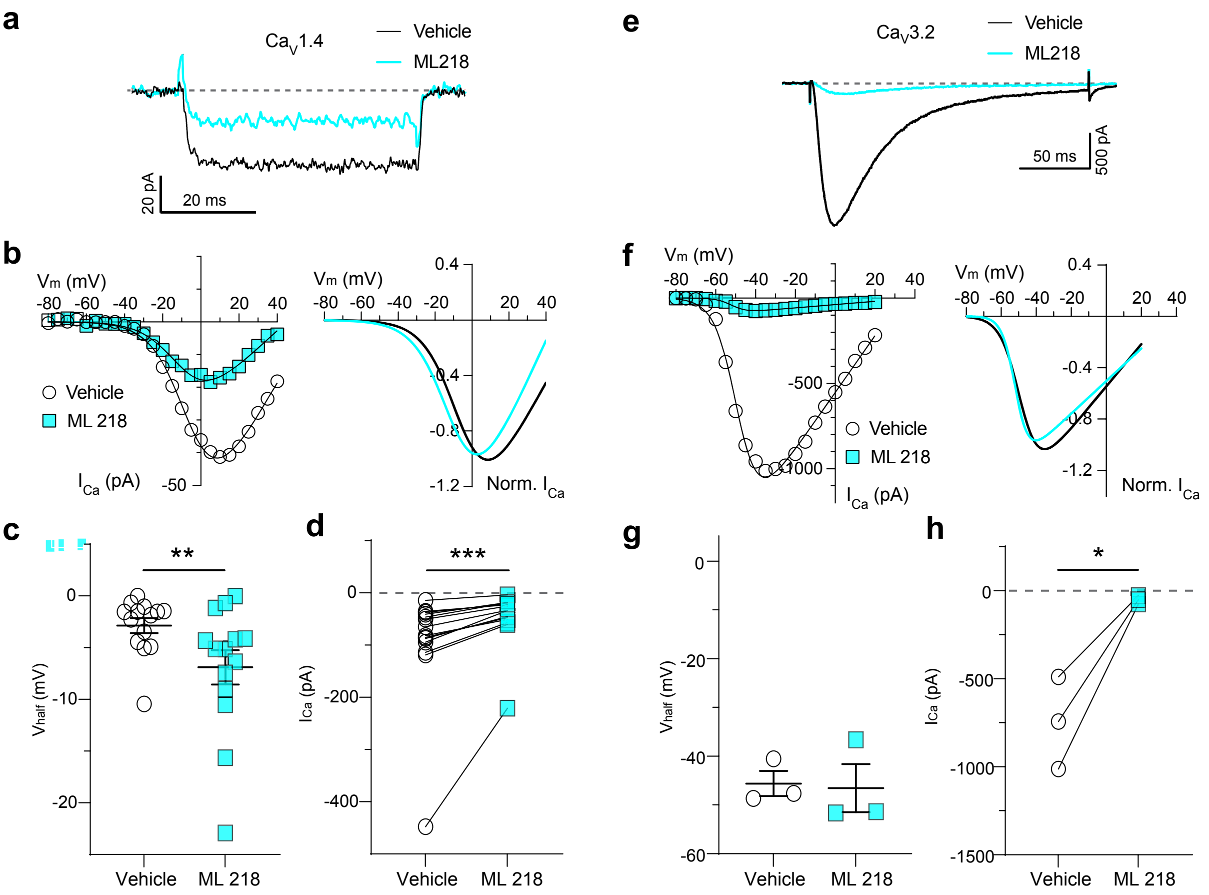

Supplementary Figure S2. Effect of 218 on Ca$_v$1.4 and Ca$_v$3.2 in EK293T cells. Cells were transfected with Ca$_V$1.4, 2x13, and 2 4 (a d) or Ca$_V$3.2 (e h). ***a,e***, epresentative traces and I V plots for I_{Ca} evoked by 50 ms steps to 10 mV (a) or 200 ms steps to 35 mV (e) from a holding voltage of 90 mV. Currents were recorded in the presence of vehicle (SO) or 218 (5 for Ca$_V$1.4 and 1 for Ca$_V$3.2). ***b,f***, eft, I V relationships for data obtained as in *a,e* but using steps to various voltages from a single representative cell. ight, olt mann fit of the responses, normali ed to the maximum I_{Ca} amplitude. ***c,d,g,h***, Graphs depicting effect of 218 on Vhalf (c,g) and I_{Ca}. ** W= 93.00, = 3.1366, p=0.001 *** W=105.0, =3.8419, p=0.0001 both by Wilcoxon matched pairs signed rank test. * t(2)=0.582, p=0.0366 by paired t test.

bioRxiv preprint doi: https://doi.org/10.1101/2023.12.05.570129; this version posted June 17, 2024. The copyright holder for this preprint (which was not certified by peer review) is the author/funder, who has granted bioRxiv a license to display the preprint in perpetuity. It is made available under aCC-BY 4.0 International license.

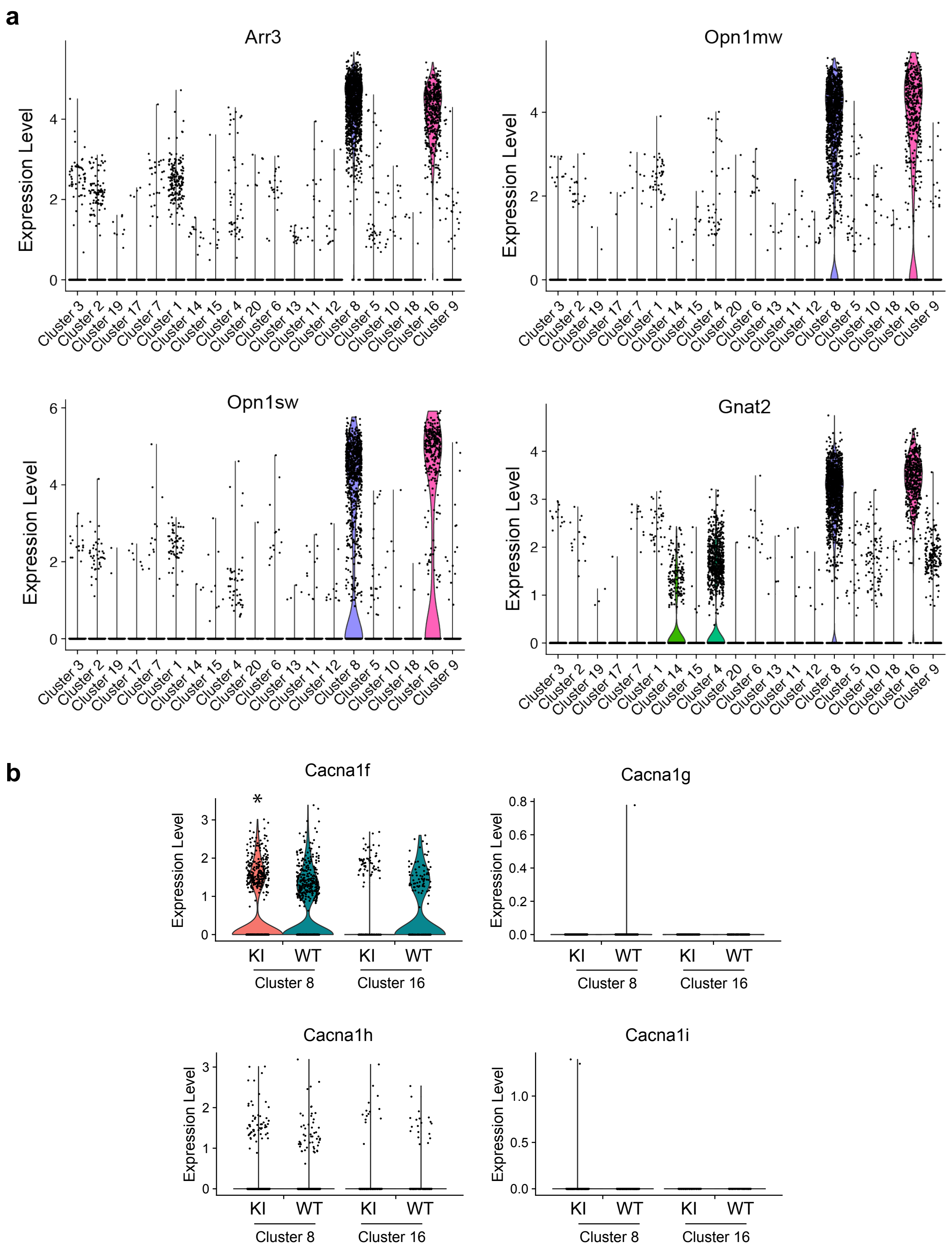

Supplementary Figure S3. Violin plots showing expression levels of transcripts at the single cell level in the retina of WT and G369i KI mice. Each point represents result from a single cell. ***a***, Expression levels of transcripts known to be expressed in cones. Cells were grouped according to their transcriptional profiles using unsupervised clustering. Clusters 8 and 16 were identified as cones based on their high expression of Arr3, Opn1mw, Opn1sw, and Gnat2. ***b***, Expression of transcripts that encode Cav1.4 (Cacna1f) and Cav3 subtypes (Cacna1g, Cacna1h, Cacna1i) in clusters 8 and 16 in retina from WT and G369i KI mice. In cluster 8, there was a small but significant decrease in the level of Cacna1f in the G369i KI vs WT (*, p = 0.01) but not in cluster 16 (p =0.09). There was no significant difference in the level of Cacna1h between genotypes in cluster 8 or 16 (p = 1).
