# Supplementary material for: A non-conducting role of the Cav1.4 Ca2+ channel drives homeostatic plasticity at the cone photoreceptor synapse
Source: bioRxiv. 2023 Dec 6:2023.12.05.570129. Preprint. [Version 1] doi: 10.1101/2023.12.05.570129 (PMC10723350; doi:10.1101/2023.12.05.570129)
Supplement: Supplementary file 1 [file NIHPP2023.12.05.570129V1-supplement-1.pdf]

bioRxiv preprint doi: https://doi.org/10.1101/2023.12.05.570129; this version posted December 6, 2023. The copyright holder for this preprint (which was not certified by peer review) is the author/funder, who has granted bioRxiv a license to display the preprint in perpetuity. It is made available under aCC-BY 4.0 International license.

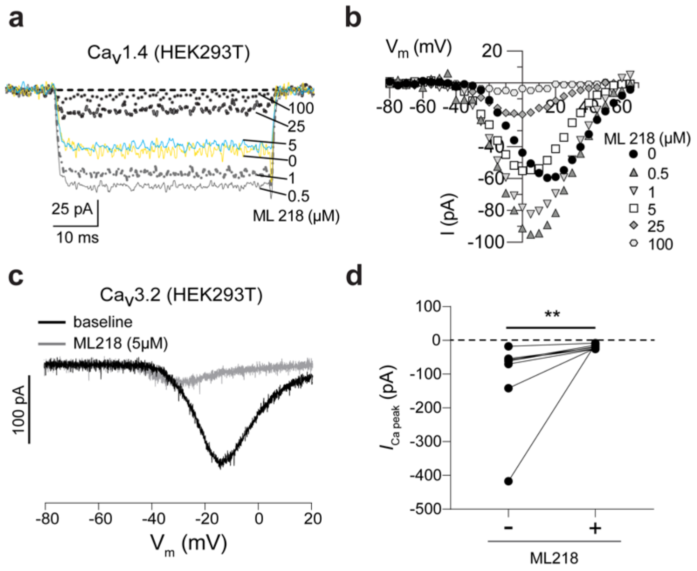

Supplementary Figure S1. Effect of ML218 on HEK293T cells transfected with $Ca_v1.4$, β_{2x13}, and $\alpha_2\delta$−4 or $Ca_v3.2$. a,b, Traces (*a*) and I-V plot (*b*) for I_{Ca} evoked by 50 ms depolarizations from -90 mv to various voltages without (0 μM) and in the presence of the indicated concentrations of ML218. **c,d**, Traces (*c*) and graph (*d*) depicting inhibitory effects of ML218 (5 μM) on $Ca_v3.2$-mediated ICa evoked by voltage ramp in individual transfected cells. **, $p < 0.01$ by paired t-test.

bioRxiv preprint doi: https://doi.org/10.1101/2023.12.05.570129; this version posted December 6, 2023. The copyright holder for this preprint (which was not certified by peer review) is the author/funder, who has granted bioRxiv a license to display the preprint in perpetuity. It is made available under aCC-BY 4.0 International license.

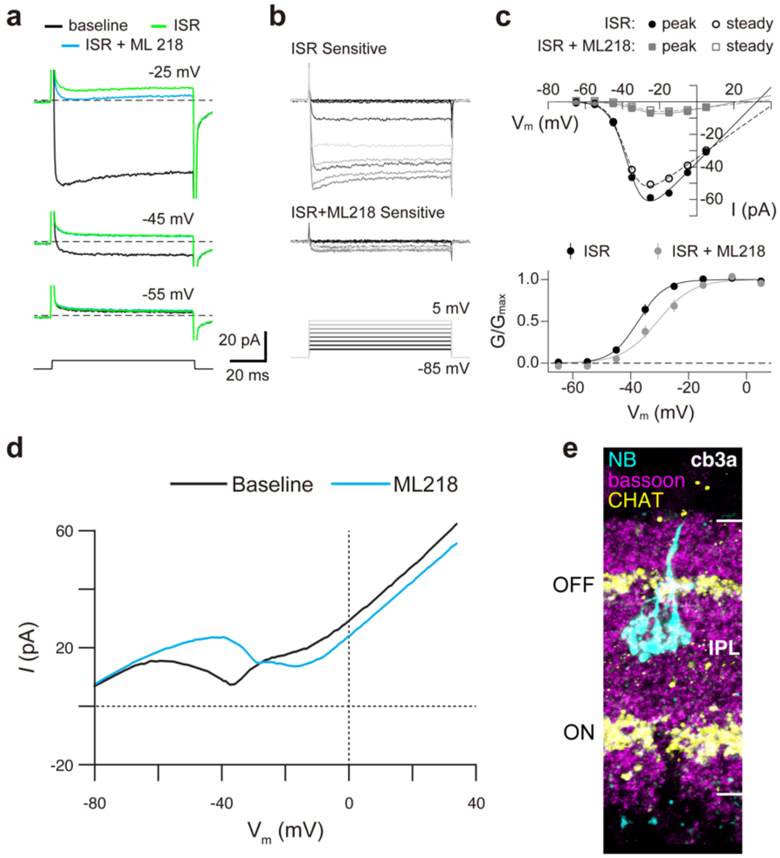

Supplementary Figure S2. Adult ground squirrel cones lack a Ca_v3-like conductance. a, Representative traces for I_{Ca} evoked by voltage steps from a continuous holding voltage of -85 mV to various voltages. Following recording of I_{Ca} bathed in control solution (baseline), cone terminals were perfused sequentially with ISR (2 µM) and then ISR (2 µM) + ML218 (5 µM). Voltage protocol is shown below current traces. Dashed line indicates zero current. **b**, Traces show the I_{Ca} sensitive to ISR (top) and ISR+ML218 (middle). I_{Ca} was evoked by steps from -85 mV to voltages between -65 and -5 mV in increments of +10 mV. The I_{Ca} recorded in ISR was subtracted from the baseline I_{Ca} (top). I_{Ca} recorded in ISR+ML218 was subtracted from I_{Ca} recorded in ISR (middle). The small amplitude and noninactivating properties of the current blocked by adding ML218 to ISR suggest that it is mediated by Cav1.4 channels that continue to undergo a time-dependent block at the concentration of ISR used here. **c**, Top, Current-voltage (I-V) plots of peak and steady-state I_{Ca} from *b*. Bottom, normalized conductance (G/G_{max}) vs. voltage relationship (G-V) of the I_{Ca} blocked by ISR and ISR + ML218. Smooth lines represent Boltzmann fits; symbols and bars represent mean ± SEM, respectively; n = 4 cones. Due a negative shift in the activation properties caused by ML218 on a residual Ca_v1 current that is assumed to remain at the end of the experiment, the G-V curve of the I_{Ca} isolated through subtraction by applying ISR+ML218 underwent a statistically insignificant shift to the right. Data presented in *a-c* are from the same cone. **d**, Voltage ramp current response in an OFF cb3a bipolar cell before and after the application of ML218 (5 µM). $V_{1/2}$ was shifted to the right by 13.1 ± 4.3 mV (n = 3 cb3a cells, mean ± SD) consistent with the block of a Ca_v3-like conductance. In this experiment, the amplitude of an I_{Ca} component that had a more depolarized activation range was slightly increased. A similar voltage ramp applied to OFF cb3b bipolar cells produced only a slight leftward shift in an I_{Ca} that had a more depolarized activation range, consistent with the exclusive expression a Ca_v1-type current ($V_{1/2}$ was shifted by -1.9 ± 0.4 mV, n = 3 cb3b cells; data not shown). **e**, Neurobiotin filled OFF cb3a. Following recordings of cells in *d*, retina slices were fixed and co-immunolabeled for bassoon and choline acetyltransferase (CHAT) to depict synapses and the OFF and ON sublamina of the innerplexiform layer (IPL), respectively. Streptavidin was used to label the neurobiotin to depict the OFF cb3a axon stratification and morphology.

bioRxiv preprint doi: https://doi.org/10.1101/2023.12.05.570129; this version posted December 6, 2023. The copyright holder for this preprint (which was not certified by peer review) is the author/funder, who has granted bioRxiv a license to display the preprint in perpetuity. It is made available under aCC-BY 4.0 International license.

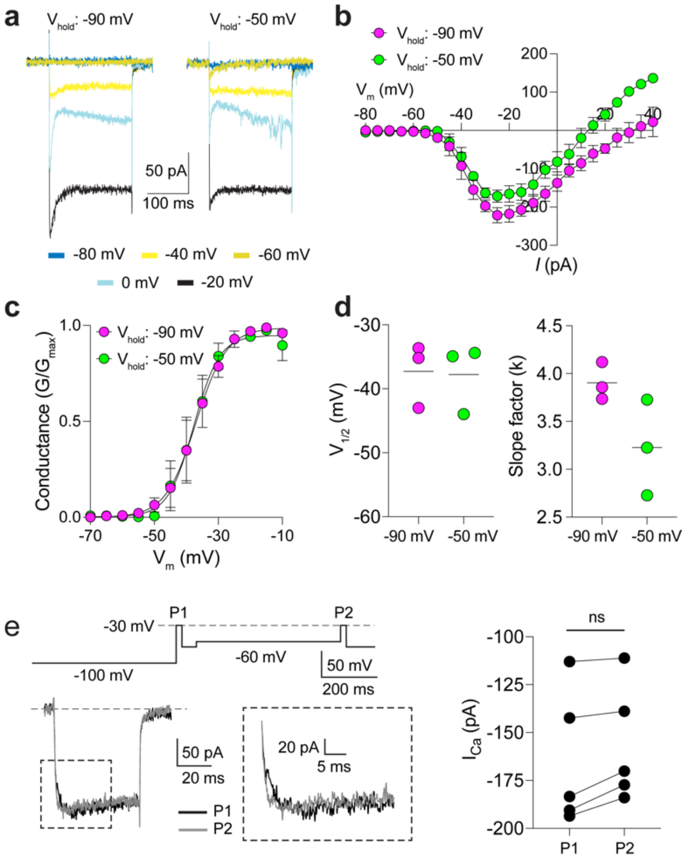

Supplementary Figure S3. Characterization of I_{Ca} in cones of macaque retina. (a-c) Representative current traces (a) and I-V (b), and G-V (b) for I_{Ca} evoked by 200-ms steps from a holding voltage (V_h) of -90 mV or -50 mV (which should isolate the Ca_v1-mediated I_{Ca} from any contribution of Ca_v3 channels). n=3 cells. (d) $V_{1/2}$ and slope factor (k) obtained from Boltzmann fit of data in *c*. There was no significant effect of holding voltage on these parameters, arguing against a contribution of Ca_v3 channels to the whole-cell I_{Ca}. (e) Lack of effect of inactivating voltage on I_{Ca}. Test currents were evoked from a holding voltage of -100 V to -30 mV before (P1) and after (P2) a 500 ms step to -60 mV. Voltage protocol shown above overlay of P1 and P2 current traces. Boxed region is shown with expanded timescale; the similar activation kinetics of P1 and P2 currents supports the contribution of a single Ca_v subtype. Graph at right shows lack of significant difference in amplitudes of P1 and P2 currents, arguing against the contribution of Ca_v3 channels to the P1 current.

bioRxiv preprint doi: https://doi.org/10.1101/2023.12.05.570129; this version posted December 6, 2023. The copyright holder for this preprint (which was not certified by peer review) is the author/funder, who has granted bioRxiv a license to display the preprint in perpetuity. It is made available under aCC-BY 4.0 International license.

Supplementary Table 1

**Male vs. Female WT (Photopic)**	SS	DF	MS	F (DFn, DFd)	P value
Trial x Sex	1125	5	224.9	F (5, 45) = 0.6060	P=0.6956
Trial	3564	5	712.9	F (5, 45) = 1.921	P=0.1096
Sex	273.6	1	273.6	F (1, 9) = 0.6616	P=0.4370
Subject	3722	9	413.5	F (9, 45) = 1.114	P=0.3727
Residual	16703	45	371.2		
**Male vs. Female G369i KI (Photopic)**	SS	DF	MS	F (DFn, DFd)	P value
Trial x Sex	1457	5	291.4	F (5, 45) = 0.5362	P=0.7477
Trial	3516	5	703.2	F (5, 45) = 1.294	P=0.2833
Sex	1150	1	1150	F (1, 9) = 0.8168	P=0.3897
Subject	12675	9	1408	F (9, 45) = 2.592	P=0.0168
Residual	24451	45	543.4		
**Male vs. Female Cav1.4 KO (Photopic)**	SS	DF	MS	F (DFn, DFd)	P value
Trial x Sex	1736	5	347.1	F (5, 35) = 0.6597	P=0.6563
Trial	993	5	198.6	F (3.056, 21.39) = 0.3775	P=0.7737
Sex	78.37	1	78.37	F (1, 7) = 0.03788	P=0.8512
Subject	14481	7	2069	F (7, 35) = 3.932	P=0.0029
Residual	18416	35	526.2		
**Male vs. Female WT (Scotopic)**	SS	DF	MS	F (DFn, DFd)	P value
Trial x Sex	862	5	172.4	F (5, 45) = 0.6907	P=0.6331
Trial	914.5	5	182.9	F (5, 45) = 0.7328	P=0.6027
Sex	634.7	1	634.7	F (1, 9) = 2.121	P=0.1793
Subject	2693	9	299.3	F (9, 45) = 1.199	P=0.3193
Residual	11232	45	249.6		
**Male vs. Female G369i KI (Scotopic)**	SS	DF	MS	F (DFn, DFd)	P value
Trial x Sex	7648	5	1530	F (5, 45) = 2.581	P=0.0390
Trial	1644	5	328.8	F (5, 45) = 0.5547	P=0.7339
Sex	4712	1	4712	F (1, 9) = 4.716	P=0.0580
Subject	8993	9	999.2	F (9, 45) = 1.686	P=0.1206
Residual	26671	45	592.7		
**Male vs. Female Cav1.4 KO (Scotopic)**	SS	DF	MS	F (DFn, DFd)	P value
Trial x Sex	1727	5	345.4	F (5, 35) = 1.470	P=0.2246
Trial	12215	5	2443	F (2.802, 19.62) = 10.40	P=0.0003
Sex	96.33	1	96.33	F (1, 7) = 0.1346	P=0.7246
Subject	5011	7	715.9	F (7, 35) = 3.046	P=0.0131
Residual	8225	35	235		
